# Hydatid Cyst Fluid of *Echinococcus granulosus* Alleviates Experimental Ulcerative Colitis by Suppressing Inflammation and Oxidative Stress and Regulating the Gut Microbiota

**DOI:** 10.3390/ijms27209286

**Published:** 2026-10-19

**Authors:** Xifeng Wang, Ayi Baota, Fei Wang, Yeergeming Didaer, Jiaoyu Shan

**Affiliations:** 1 Department of Human Parasitology, School of Basic Medical Sciences, Xinjiang Medical University, Urumqi 830011, China; 2 Xinjiang Key Laboratory of Molecular Biology for Endemic Diseases, Urumqi 830011, China

**Keywords:** *Echinococcus granulosus*, HCF, ulcerative colitis, Keap1/Nrf2, Gut microbiota, metabolites

## Abstract

Ulcerative colitis (UC) is a chronic recurrent inflammatory disease of the colonic mucosa, with a rising global incidence. Hydatid cyst fluid of *Echinococcus granulosus* (EgHCF) has been shown to alleviate UC inflammation, but how it exerts this protective effect is not yet fully understood. In this study, we investigated the protective efficacy of EgHCF using a dextran sulfate sodium (DSS)-induced acute colitis model in mice and an NCM460 intestinal epithelial cell line. In vivo, EgHCF treatment significantly ameliorated the clinical signs of DSS-induced colitis, counteracted colon shortening and weight loss, and mitigated colonic histopathological damage; furthermore, it enhanced intestinal barrier integrity, suppressed inflammatory responses, reduced oxidative stress, and activated the Keap1/Nrf2 signaling pathway. Gut microbiota analysis revealed that EgHCF attenuated gut microbial dysbiosis by increasing α-diversity and the abundance of *Firmicutes* and *Verrucomicrobiota*. Metabolomics analysis further indicated that the tryptophan metabolic pathway plays a prominent role in the treatment of DSS-induced UC mice with EgHCF. In vitro, EgHCF protected the epithelial barrier function of NCM460 cells, significantly inhibited the expression of pro-inflammatory factors iNOS and COX-2, attenuated intracellular ROS levels, and activated the Keap1/Nrf2 pathway in DSS-stimulated NCM460 cells. These findings suggest that EgHCF alleviates DSS-induced colitis by mitigating inflammation and oxidative stress, as well as regulating the gut microbiota, providing novel insights into its role in repairing the intestinal barrier.

## 1. Introduction

Inflammatory bowel disease (IBD), a chronic inflammatory disorder of the gastrointestinal tract encompassing Crohn’s disease (CD) and ulcerative colitis (UC), is characterized by persistent mucosal inflammation and disruption of the intestinal epithelial barrier [1]. The pathogenesis of IBD is multifactorial and complex, and emerging evidence indicates that these diverse etiological factors are closely interconnected through the gut microbiome [2]. Currently, conventional treatments, including 5-aminosalicylic acid (5-ASA), corticosteroids, and immunomodulators, remain effective options for the treatment of UC [3]. In recent years, the therapeutic armamentarium for IBD has broadened considerably with the advent of agents operating through diverse mechanistic pathways, including targeted biologics and small-molecule inhibitors [4]. Despite these advances, a significant proportion of patients still experience treatment resistance or incomplete responses [5]. Consequently, a critical unmet need remains for the development of safer and more effective treatment strategies for UC.

Helminthiasis imposes a substantial disease burden on both human and animal health. Intestinal helminth infection induces profound alterations in the composition and function of the host gut microbiota, and helminths can also modulate microbial communities at distant anatomical sites beyond the gastrointestinal tract [6,7]. Cystic echinococcosis (CE), one of the most significant parasitic zoonoses worldwide, is caused by the larval stage (hydatid cyst) of *Echinococcus granulosus* sensu lato (Eg). The hydatid wall consists of two main layers from the outside to inside: the laminated layer (LL) and the germinal layer (GL). The LL is a carbohydrate-rich structure derived from the parasite that plays a crucial role in modulating host immune responses [8]; the GL, a nucleated and metabolically active cellular layer, produces brood capsules and protoscoleces (PSCs) and also secretes hydatid cyst fluid (HCF) [9]. EgHCF, a clear-to-yellow fluid that fills the entire cyst, exhibits antigenic properties and provides nutrients for larval growth [10]. Notably, recent evidence indicates that it enhances the protective effect of sulfasalazine against murine colitis by upregulating anti-inflammatory and antioxidant defenses [11]. However, whether EgHCF can also alleviate colitis through regulating inflammatory response and oxidative stress, and how it regulates the gut microbiota and metabolites, remain poorly understood.

Therefore, this study aimed to systematically investigate the protective efficacy of EgHCF in DSS-induced UC mice and its effects on the host gut microbiota and metabolites. Using a DSS-induced UC mice model and an NCM460 cell model, we evaluated its effects on inflammation, oxidative stress, and the Keap1/Nrf2 signaling pathway. Furthermore, EgHCF modulatory effects on the gut microbiota and metabolites were assessed via 16S rRNA sequencing and untargeted metabolomics. This study provides a foundational basis for future investigations into the role of EgHCF.

## 2. Results

### 2.1. EgHCF Attenuated DSS-Induced UC in Mice

**Figure 1:**
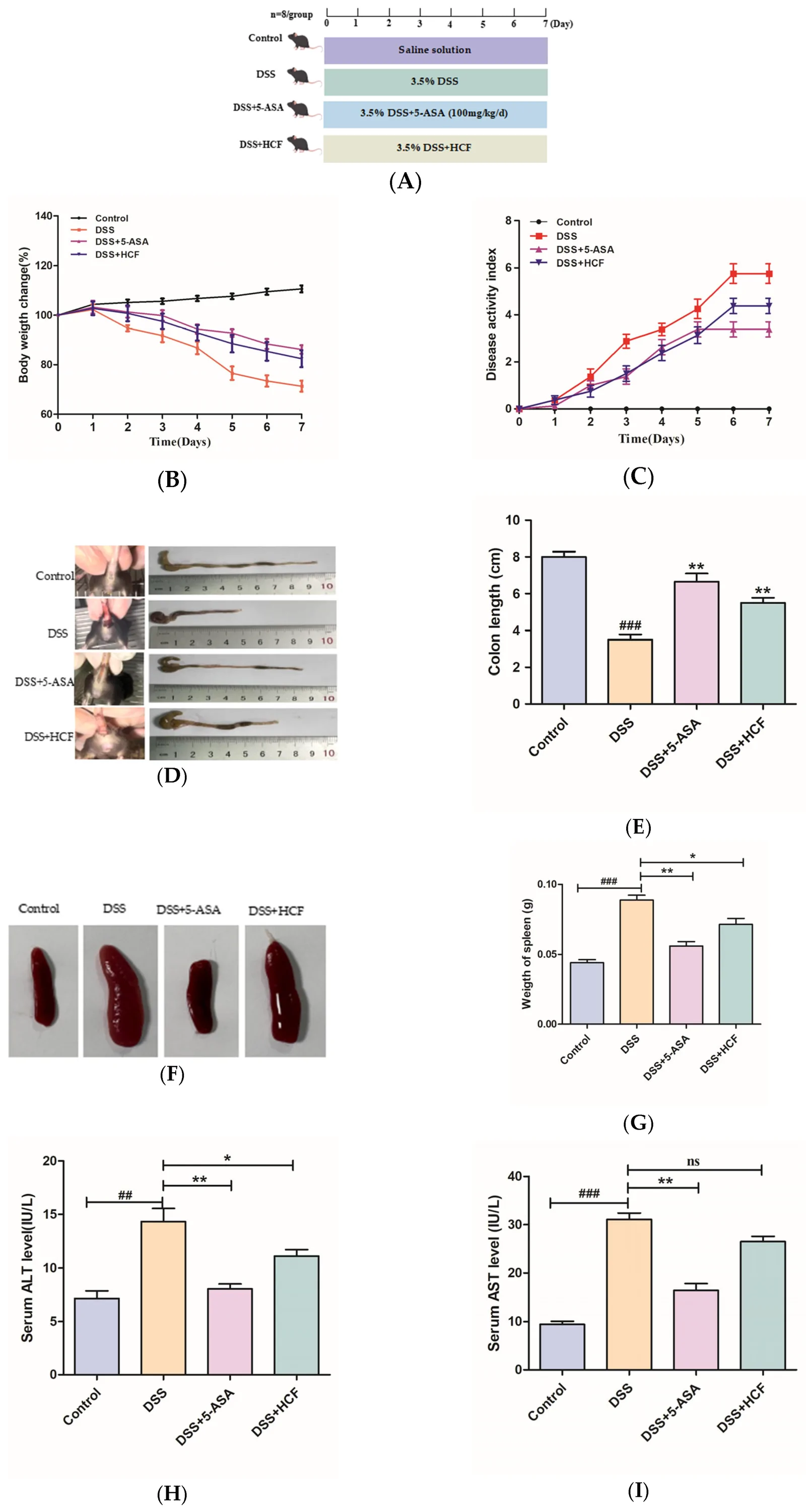

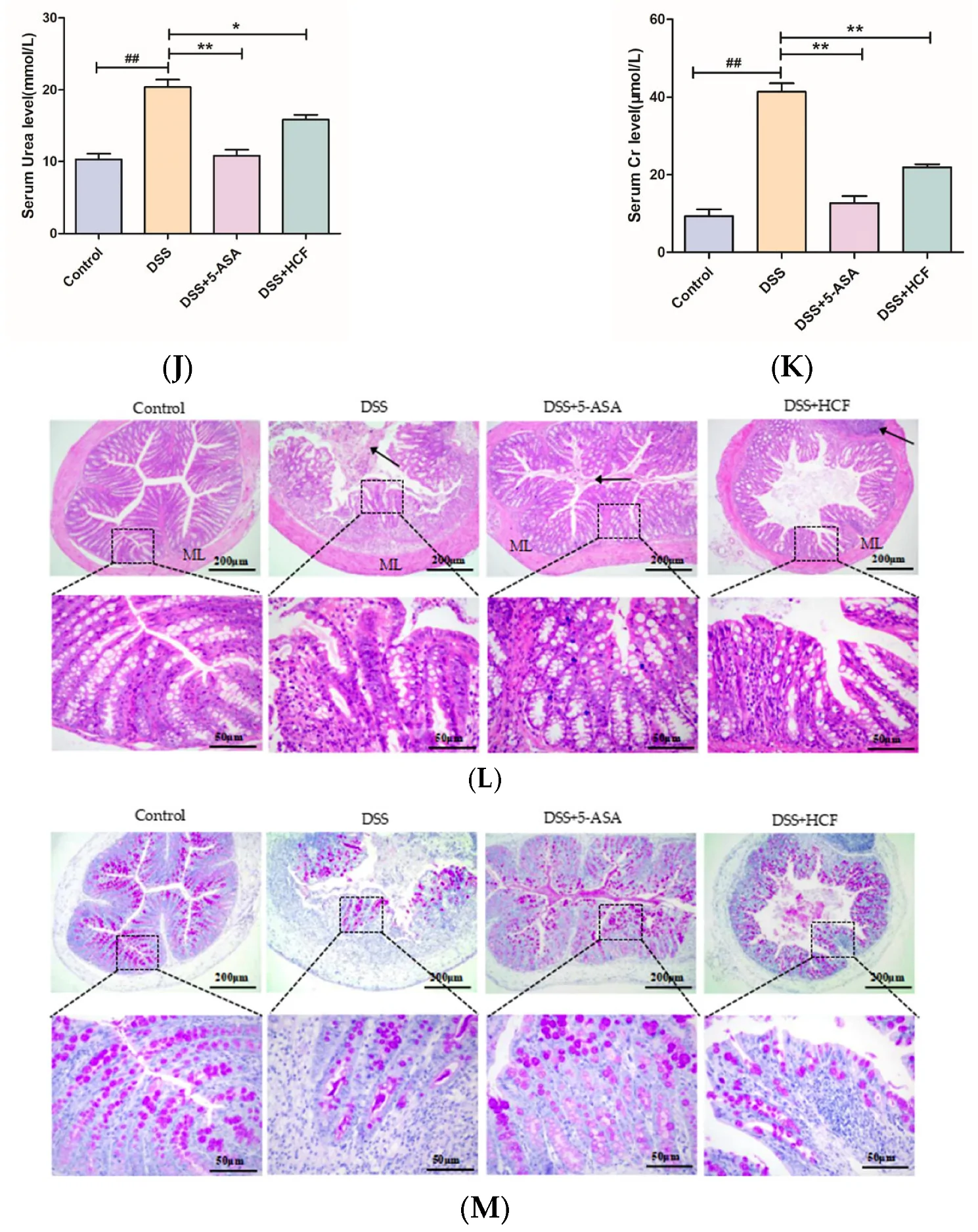
EgHCF attenuated DSS-induced UC in mice. (**A**) The schematic design of the animal experiment. (**B**) Relative values of body weight changes in mice. (**C**) The changes in disease activity index of each group during the trial period. (**D**) Representative images of perianal bleeding and appearance of the colon in each group of mice. (**E**) Length of colons. (**F**) Representative images of spleens in mice. (**G**) Spleen weight of mice. (**H**,**I**) Serum ALT and AST levels in mice. (**J**,**K**) Serum urea and creatinine concentrations in mice. (**L**) Representative photographs of H&E staining of colon tissues in each group. The infiltration of inflammatory cells is represented by black arrows. ML: muscular layer. (**M**) Periodic Acid-Schiff stain. Data are presented as mean ± SD (*n* = 8 per group). *^##^ p* < 0.01, and *^###^ p* < 0.001 versus the control group; * *p* < 0.05, and ** *p* < 0.01, versus the DSS group. “ns” represents no significant difference.

To investigate the regulatory role of EgHCF in UC, a DSS-induced colitis model was established in mice. The detailed animal experimental procedure is illustrated in Figure 1A. Compared with the control group, DSS-treated mice showed significant body weight loss; however, both EgHCF and 5-ASA treatments effectively reversed this reduction (Figure 1B). Similarly, mice treated with EgHCF or 5-ASA exhibited attenuated DAI scores compared with those in the DSS group (Figure 1C). Colon shortening, a hallmark of colitis, was prominent in DSS-treated mice (*p* < 0.001 versus control); however, this reduction was notably recovered by EgHCF or 5-ASA (Figure 1D,E). In addition, both treatments significantly alleviated the DSS-induced splenomegaly (Figure 1F,G).

Levels of serum alanine aminotransferase (ALT) (Figure 1H) and aspartate aminotransferase (AST) (Figure 1I), two biochemical indicators of liver damage, were markedly elevated in the DSS group; 5-ASA treatment restored both parameters back to near-normal levels, whereas EgHCF significantly reduced ALT but exerted only a modest effect on AST. Furthermore, DSS-treated mice exhibited higher serum urea (Figure 1J) and creatinine (Cr) (Figure 1K) levels than controls, and both 5-ASA and EgHCF significantly mitigated these markers of renal impairment associated with UC modeling.

H&E staining showed that the colonic mucosal epithelium in the control group remained structurally intact, with abundant goblet cells. In contrast, the DSS group exhibited marked submucosal disruption, extensive inflammatory cell infiltration, irregular crypt size and architecture, disorganized arrangement, and a pronounced reduction in goblet cell numbers. Notably, both EgHCF and 5-ASA treatments ameliorated colonic tissue injury in mice, characterized by reduced inflammatory cell infiltration, along with restoration of crypt architecture and goblet cell numbers (Figure 1L). Consistently, periodic acid-schiff (PAS) staining showed that the DSS group exhibited substantial goblet cell depletion and a thinner mucus layer, and these abnormalities were effectively reversed following EgHCF treatment (Figure 1M).

In summary, these results demonstrated that EgHCF attenuated DSS-induced ulcerative colitis in mice.

### 2.2. EgHCF Attenuated Intestinal Barrier Damage in UC Mice

**Figure 2:**
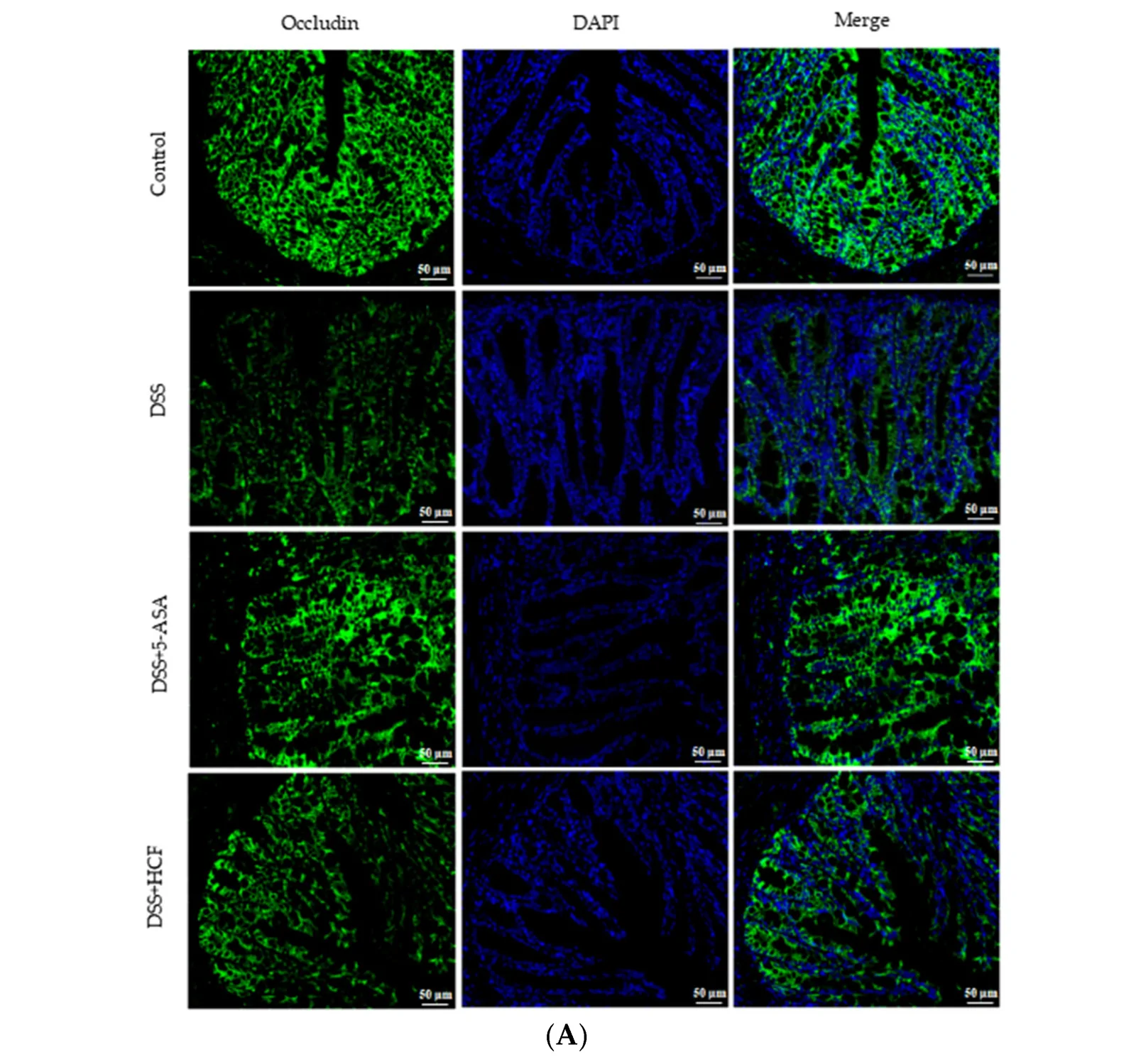

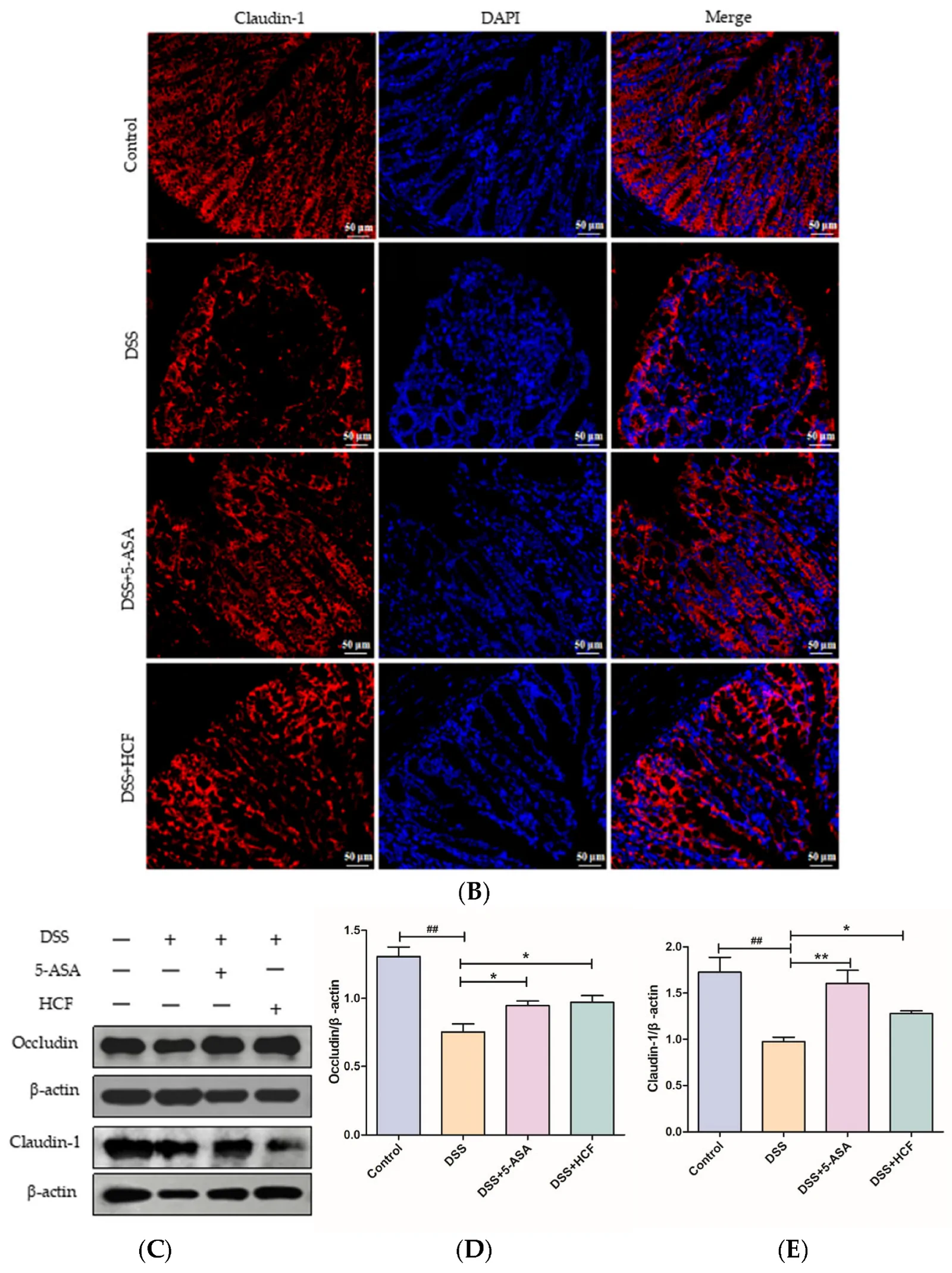
EgHCF attenuated DSS-induced intestinal barrier damage in UC mice. The expression levels of tight junction proteins (**A**) occludin (green fluorescence) and (**B**) Claudin-1 (red fluorescence) were observed by immunofluorescence staining, with cell nuclei stained blue. The merged images show the overlay. (**C**) Western blot analysis of the expression levels of occludin and Claudin-1. (**D**,**E**) Quantitative analysis of occludin and Claudin-1 expression levels normalized to β-actin. Data are presented as mean ± SD (*n* = 3). *^##^ p* < 0.01 versus the control group; * *p* < 0.05 and ** *p* < 0.01 versus the DSS group.

Tight junction (TJ) proteins are critical constituents of the intestinal barrier that regulate intestinal permeability and maintain the structural and functional integrity of the physical barrier. Reduced expression of TJ proteins has been directly associated with intestinal barrier damage [12]. Accordingly, to evaluate the protective effects of EgHCF on the intestinal barrier in mice with colitis, immunofluorescence (IF) staining and Western blot analysis were employed to assess the expression of TJ proteins in intestinal tissues. IF analysis revealed that, compared with the control group, the relative fluorescence intensity of occludin and Claudin-1 in colonic tissues was significantly decreased in the DSS-treated group; however, treatment with EgHCF or 5-ASA effectively reversed this alterations (Figure 2A,B). Consistently, Western blot analysis demonstrated that the protein expression levels of occludin and Claudin-1 were markedly downregulated in the colon tissues of DSS-treated mice, and this reduction was reversed following EgHCF treatment (Figure 2C–E). Collectively, these findings indicate that EgHCF confers a protective effect against intestinal barrier damage in DSS-induced UC mice.

### 2.3. EgHCF Reduced the Inflammatory Response in DSS-Induced UC Mice

**Figure 3:**
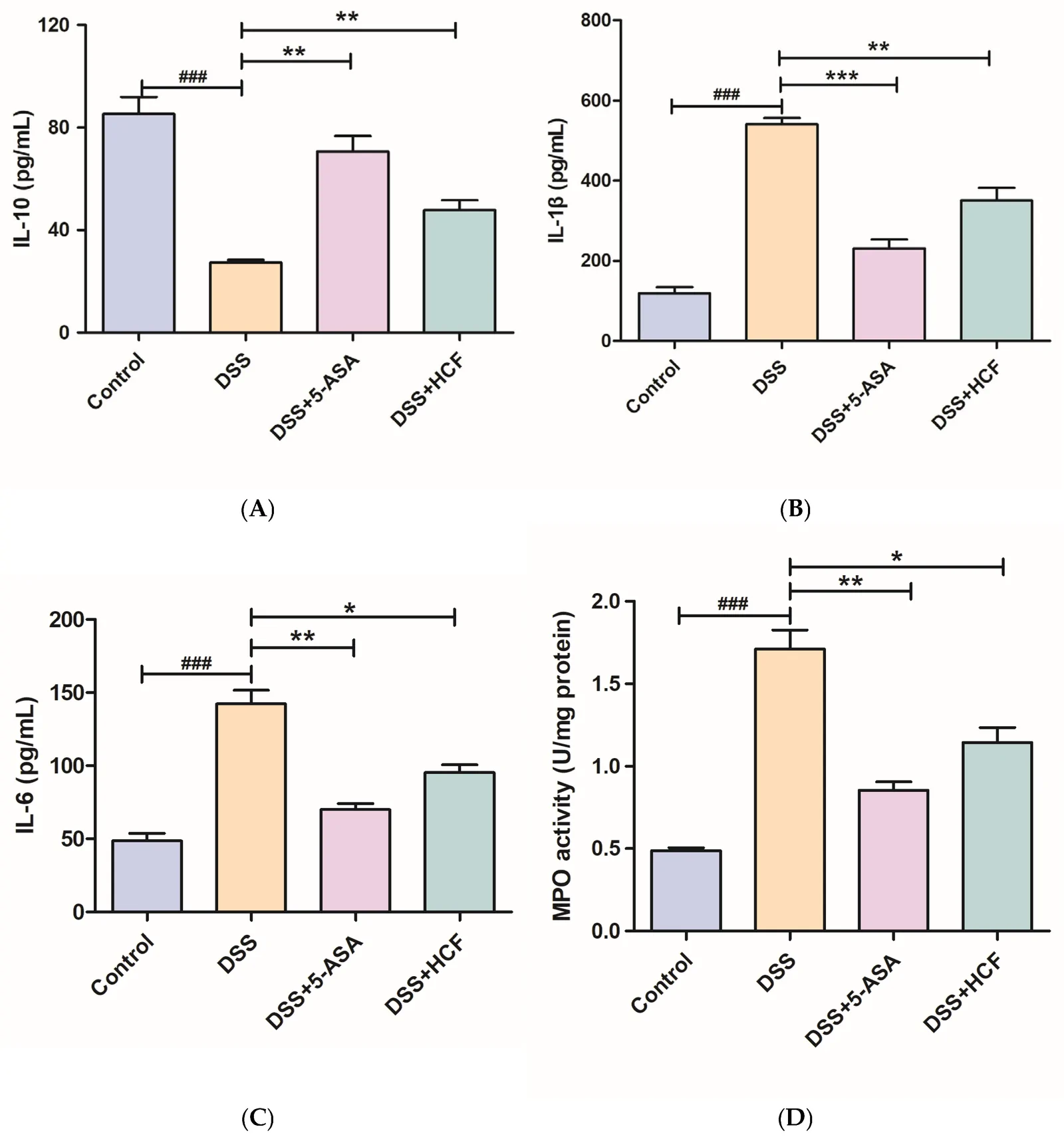

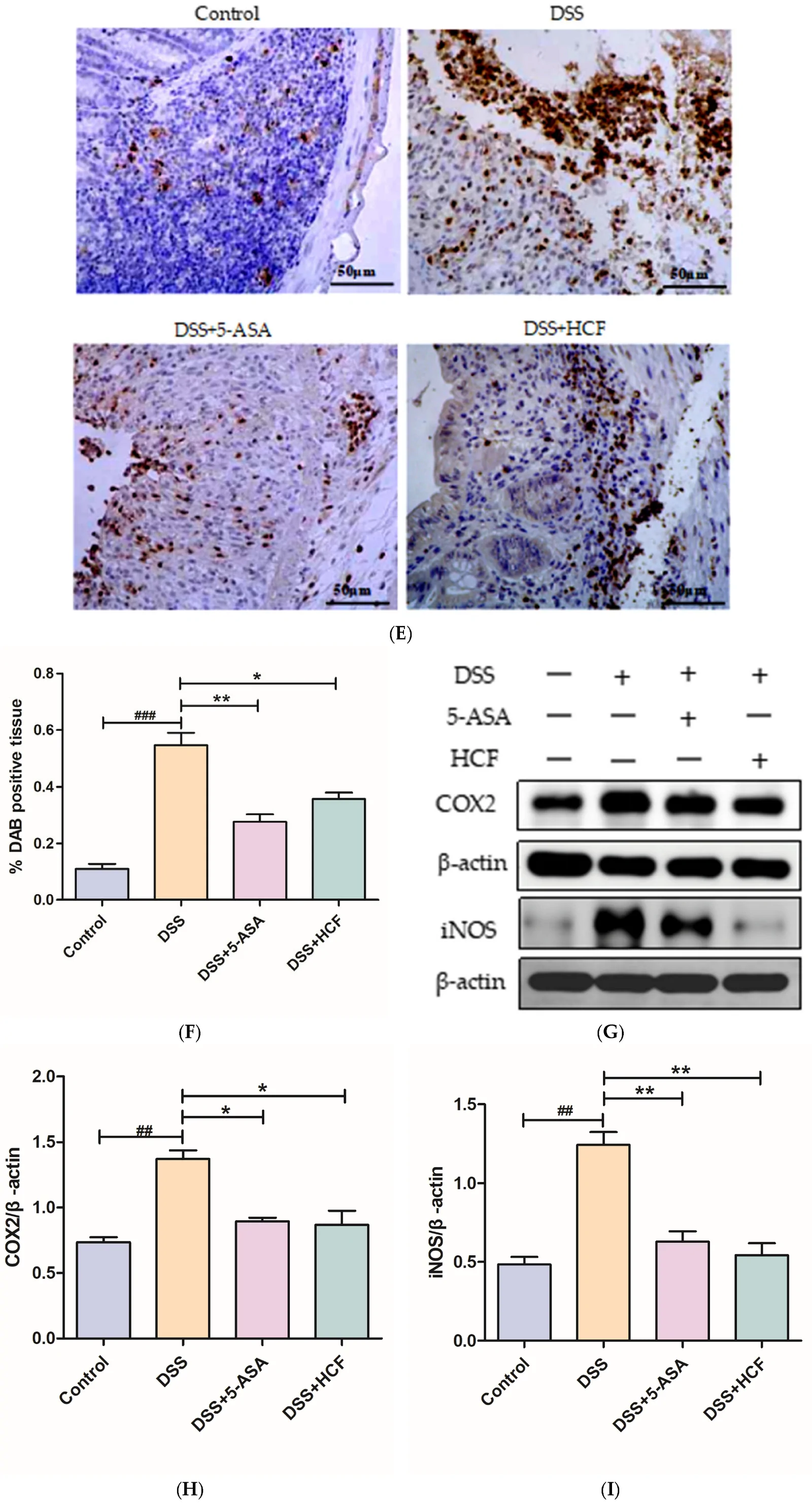
EgHCF suppressed inflammation in DSS-induced UC mice. (**A**–**C**) IL-10, IL-1β, and IL-6 levels in serum were determined by ELISA. (**D**) Activity of MPO in colon tissues was determined by ELISA. (**E**) Immunohistochemical detection of MPO expression in colon tissue sections. (**F**) Quantitative analysis of MPO expression. (**G**) The expression levels of COX2 and iNOS were detected by Western blot. (**H**,**I**) Quantitative analysis of COX2 and iNOS expression levels normalized to β-actin. Data are presented as mean ± SD (*n* = 3). *^##^ p* < 0.01, *^###^ p* < 0.001 versus the control group; * *p* < 0.05, ** *p* < 0.01, *** *p* < 0.001 versus the DSS group.

The inflammatory response is driven by an imbalance between excessive secretion of pro-inflammatory cytokines and insufficient production of anti-inflammatory cytokines, which play a critical role in the pathogenesis of ulcerative colitis [13]. To explore the effects of EgHCF on inflammation in UC mice, we measured the levels of inflammatory cytokines in serum. ELISA results showed that, compared with the control group, the DSS group exhibited markedly elevated levels of IL-1β and IL-6 and significantly reduced IL-10 levels. Notably, treatment with EgHCF significantly suppressed the DSS-induced upregulation of pro-inflammatory cytokines while substantially restoring IL-10 levels (Figure 3A–C).

As shown in Figure 3D, myeloperoxidase (MPO) activity, a key indicator of neutrophil infiltration, was significantly elevated in the DSS group (1.71 ± 0.202 U/g), whereas a marked decrease was observed in EgHCF-treated mice (1.143 ± 0.156 U/g) relative to the DSS group (Figure 3D). Consistently, immunohistochemistry (IHC) analysis revealed that MPO expression was substantially increased in the DSS group, while EgHCF treatment effectively reduced this expression (Figure 3E,F). Similarly, the protein expression levels of COX-2 and iNOS were significantly upregulated in the DSS group relative to the control group, and notably, EgHCF treatment markedly attenuated this upregulation (Figure 3G–I). These results demonstrate that EgHCF alleviates inflammatory responses in DSS-induced UC mice.

### 2.4. EgHCF Alleviates Oxidative Stress in DSS-Induced UC Mice

**Figure 4:**
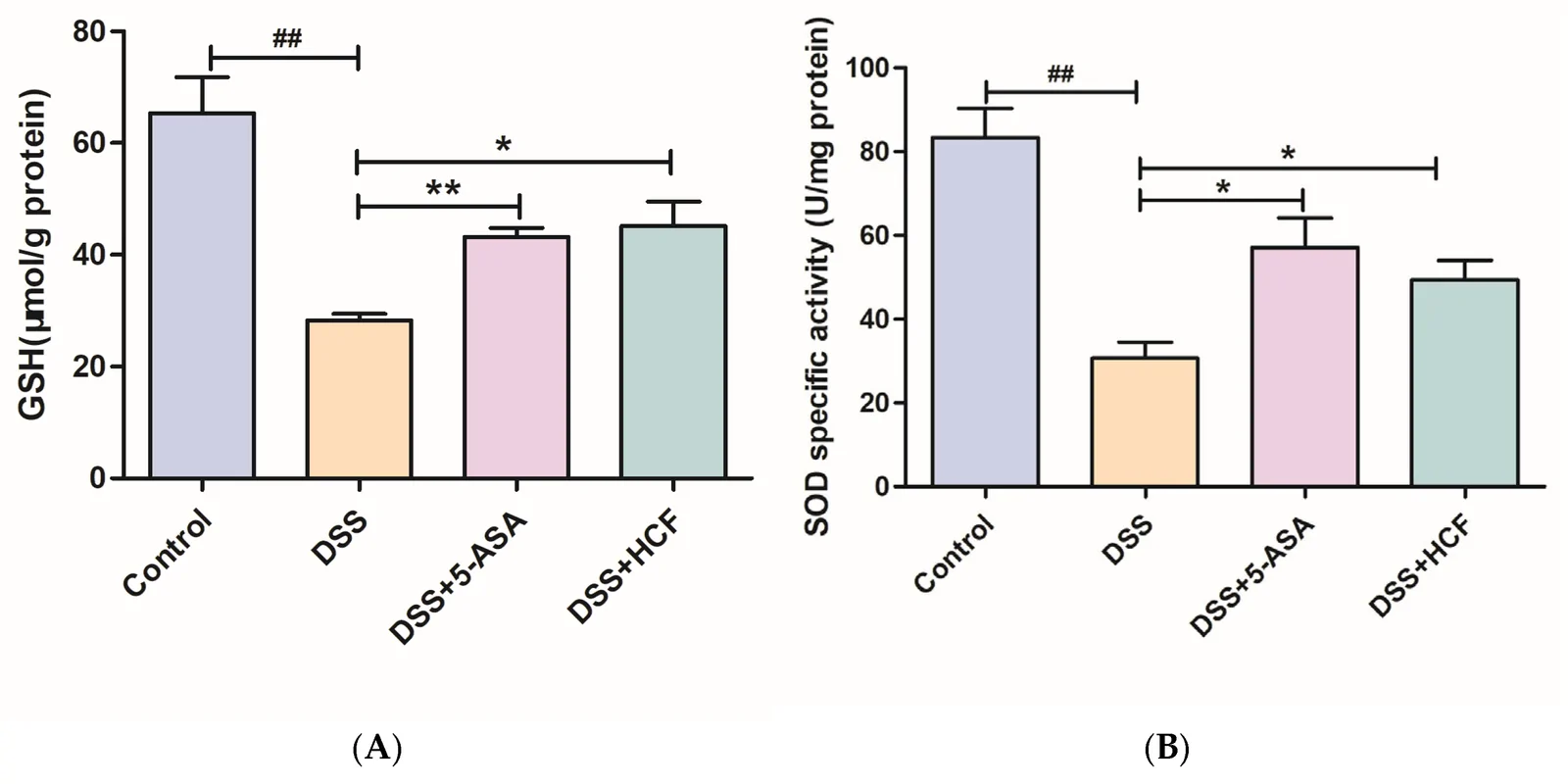

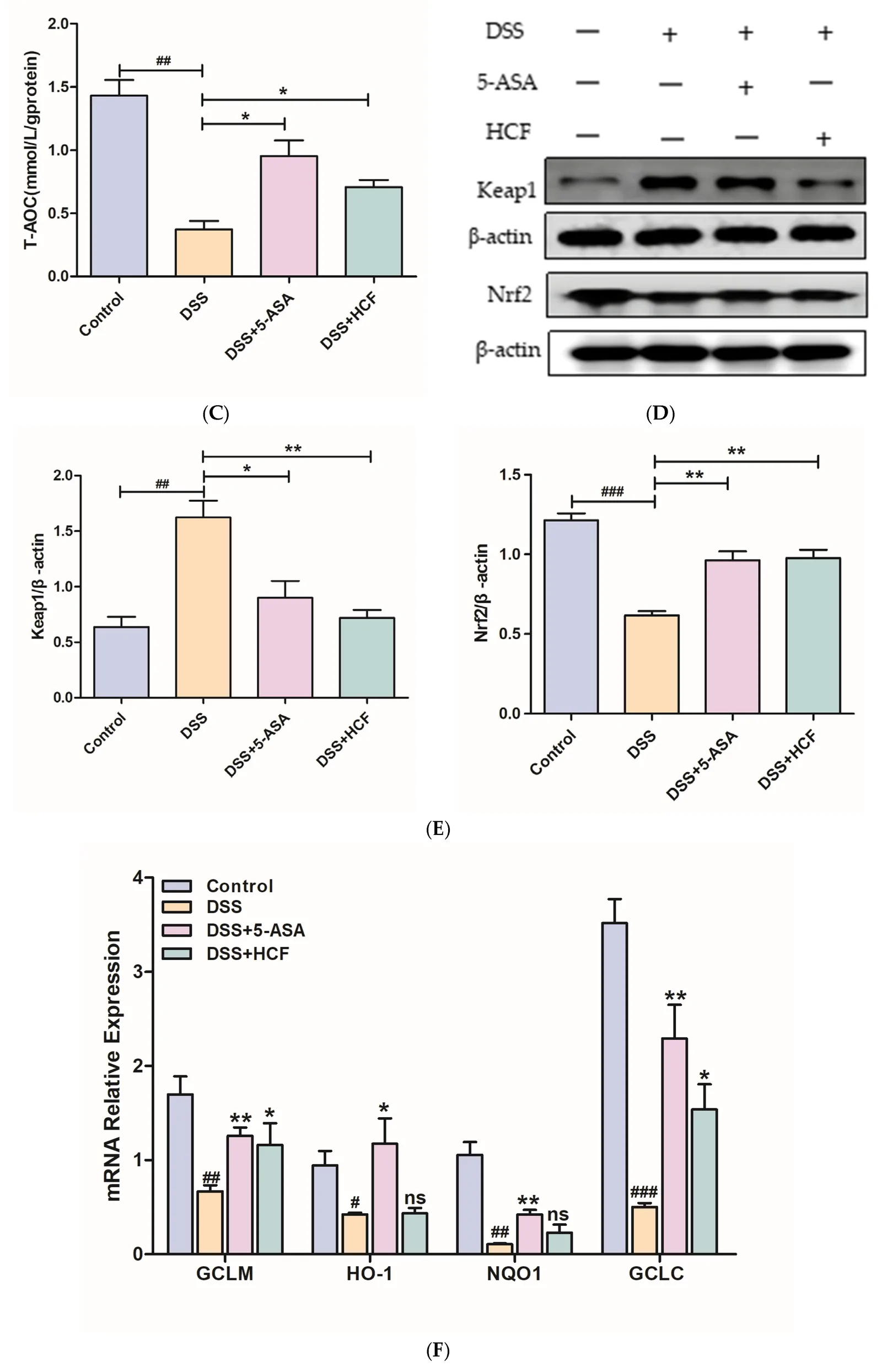
EgHCF activated the Keap1/Nrf2 signaling pathway and upregulated the mRNA expression of Nrf2-dependent downstream genes. (**A**–**C**) Measurement of the levels of oxidative stress indexes GSH, SOD and T-AOC in colon tissue. (**D**,**E**) The expression levels of Keap1 and Nrf2 were detected by Western blot, and quantitative analysis normalized to β-actin. (**F**) The mRNA expression levels of GCLM, HO-1, NQO1 and GCLC. Data are shown as mean ± SD (*n* = 3). *^#^ p* < 0.05, *^##^ p* < 0.01, *^###^ p* < 0.001 versus the control group; * *p* < 0.05 and ** *p* < 0.01 versus the DSS group. “ns” represents no significant difference.

The Kelch-like ECH-associated protein 1 (Keap1)/Nuclear factor erythroid 2-related factor 2 (Nrf2) pathway, serving as the major sensor of cellular oxidative stress, is crucial for maintaining intestinal barrier function and restraining excessive inflammatory processes [14]. To evaluate the effect of EgHCF on oxidative stress in DSS-induced UC mice, we measured the levels of glutathione (GSH), superoxide dismutase (SOD), and total antioxidant capacity (T-AOC). Compared with the control group, the DSS group exhibited significantly decreased GSH and T-AOC levels, as well as reduced SOD activity (*p* < 0.01), while treatment with EgHCF or 5-ASA markedly restored these parameters (Figure 4A–C). Western blot analysis further revealed that DSS treatment upregulated Keap1 protein expression while downregulating Nrf2 expression relative to the control group, and these alterations were significantly reversed by EgHCF treatment (Figure 4D,E). In addition, quantitative real-time PCR (qRT-PCR) analysis revealed that DSS treatment markedly downregulated the mRNA expression of Nrf2 downstream genes GCLM, HO-1, NQO1, and GCLC compared with the control group. Treatment with 5-ASA significantly restored the transcriptional levels of all four genes, whereas EgHCF upregulated GCLM and GCLC mRNA expression but exerted no significant effect on HO-1 and NQO1 (Figure 4F).

### 2.5. EgHCF Modulated Gut Microbial Diversity in DSS-Induced UC Mice

**Figure 5:**
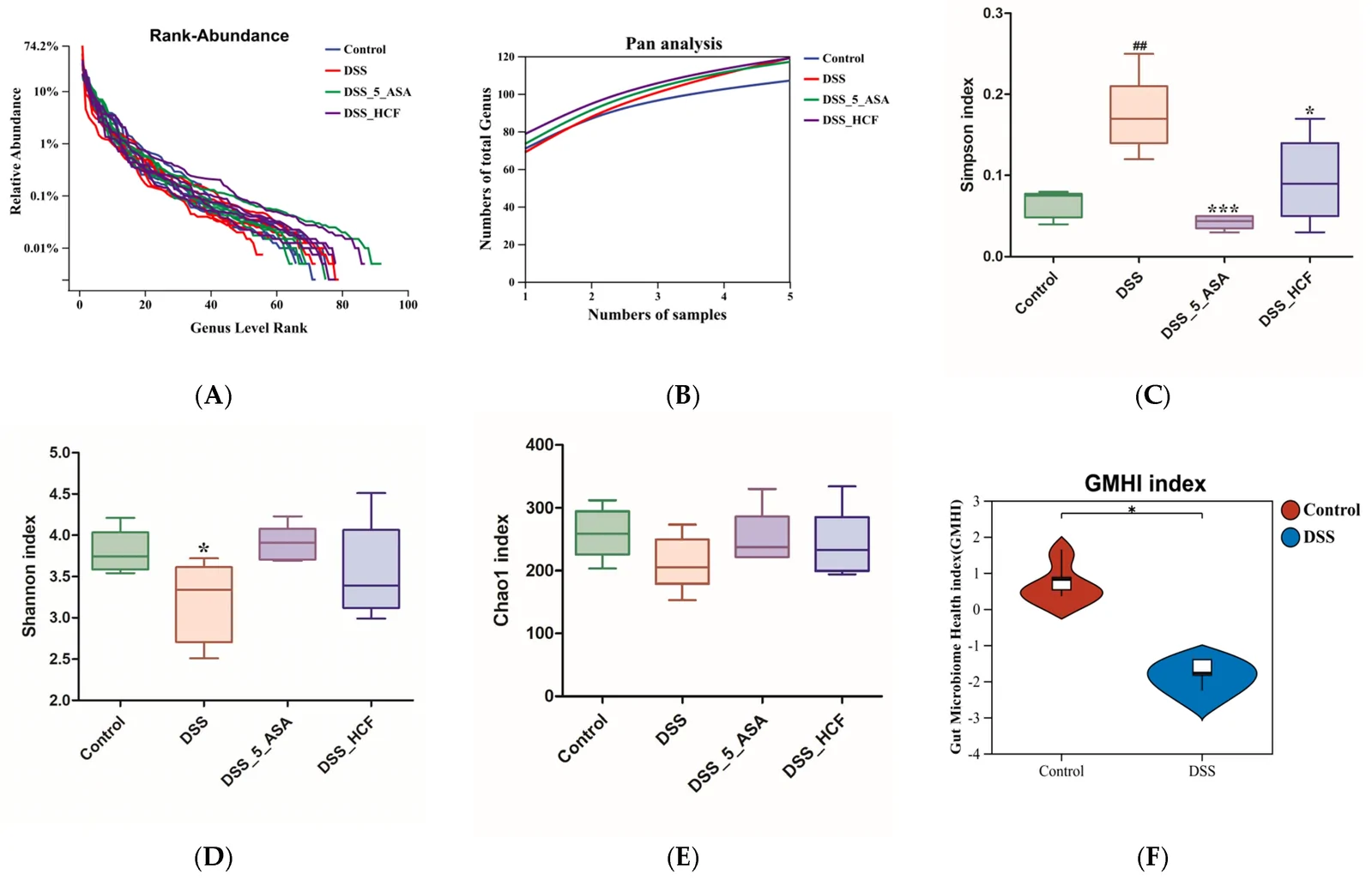

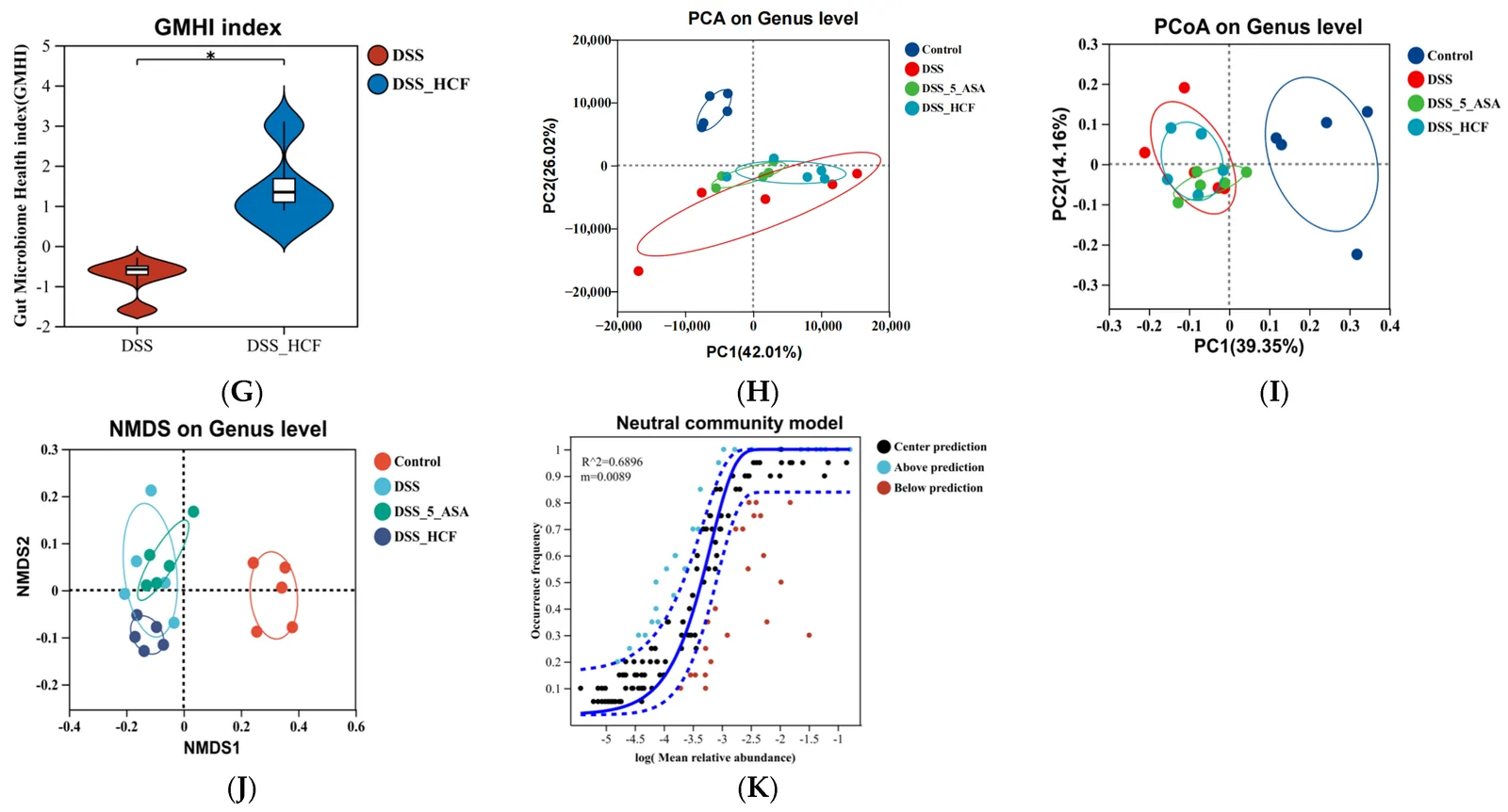
The effect of EgHCF on gut microbial diversity in DSS-induced UC mice. (**A**) Rank– abundance curve. (**B**) Pan analysis. (**C**–**E**) α–diversity analysis of the intestinal microflora for Simpson, Shannon, and Chao1 index analysis. (**F**,**G**) Gut microbiome health index analysis. (**H**) PCA. (**I**) PCoA. (**J**) NMDS. (**K**) Neutral community model analysis. Data are shown as mean ± SD (*n* = 5). *^##^ p* < 0.01 versus the control group; * *p* < 0.05, *** *p* < 0.001 versus the DSS group.

In animal models of IBD, helminth infection attenuates intestinal inflammation through multiple immune regulation mechanisms, while the composition of the host gut microbiota adapts to environmental changes during infection [15,16]. To explore how EgHCF influences gut microbiota diversity in DSS-induced UC mice, 16S rRNA high-throughput sequencing was performed across all groups. Rank-abundance curves and Pan analysis demonstrated that both the sequencing depth and the gut microbial diversity in each group were adequate for downstream analyses (Figure 5A,B).

α-diversity analysis showed that the Simpson index was markedly elevated in the DSS group relative to controls; however, EgHCF treatment significantly attenuated this increase (Figure 5C). Conversely, the Shannon index was significantly reduced in DSS-treated mice, but was restored following EgHCF administration (Figure 5D). The Chao1 index also decreased in the DSS group, and showed a modest increase with EgHCF treatment, although this difference did not reach statistical significance (Figure 5E). Furthermore, the gut microbiota health index (GMHI) was markedly lower in the DSS group than in controls, and this reduction was significantly ameliorated by EgHCF treatment (Figure 5F,G).

Principal component analysis (PCA), principal coordinate analysis (PCoA), and non-metric multidimensional scaling (NMDS) analyses were performed to assess β-diversity of the gut microbiota. These analyses revealed that the microbial composition in the control and EgHCF groups was significantly distinct from that in the DSS group (Figure 5H–J). The neutral community model (NCM) analysis showed an overall goodness-of-fit R^2^ of 0.6896, suggesting that stochastic processes played a dominant role in shaping the microbial community composition (Figure 5K). Collectively, these results demonstrate that EgHCF can modulate the structure of gut microbiota and effectively alleviate DSS-induced gut microbial dysbiosis in mice.

### 2.6. EgHCF Regulated Gut Microbiota in DSS-Induced UC Mice

**Figure 6:**
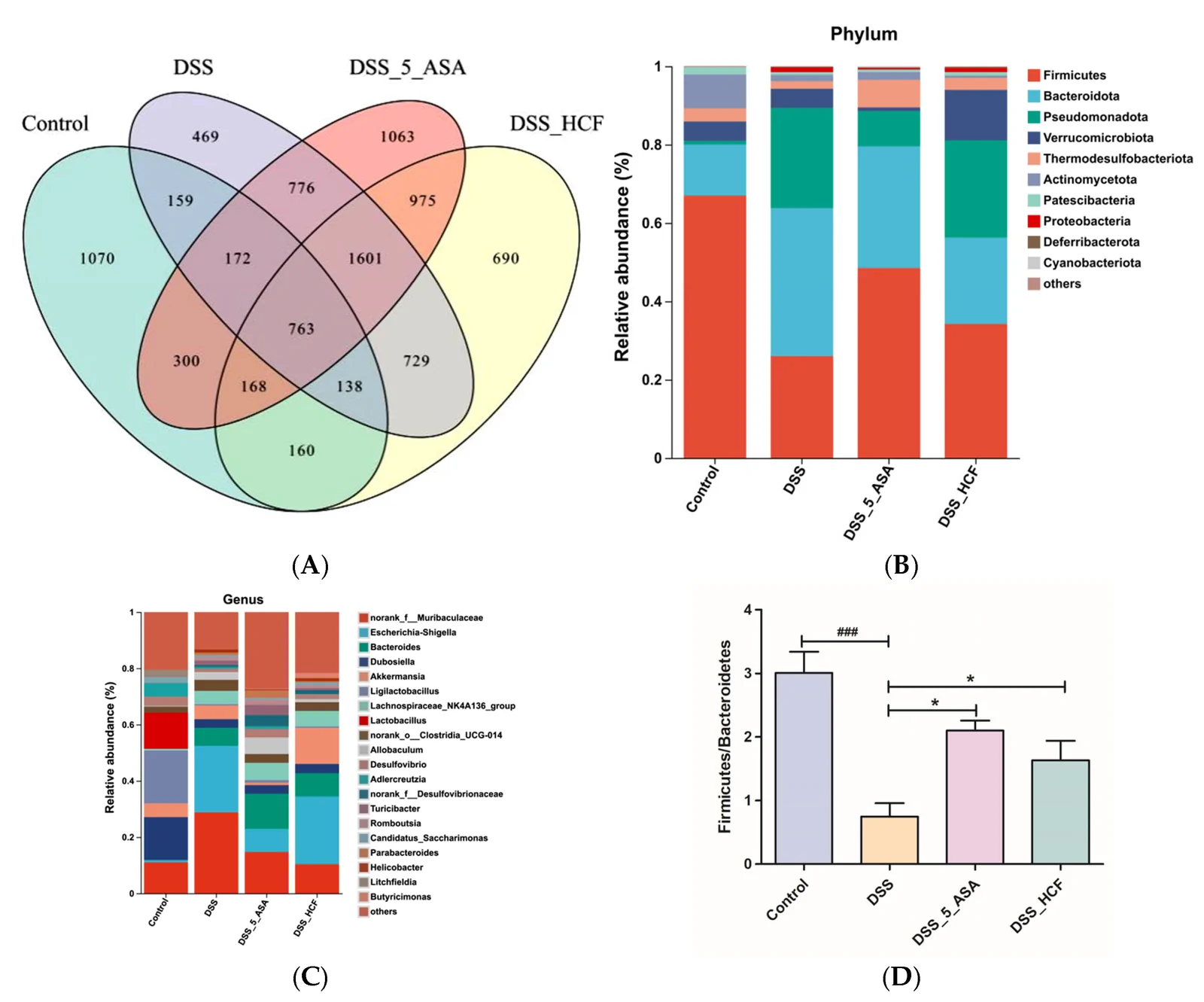

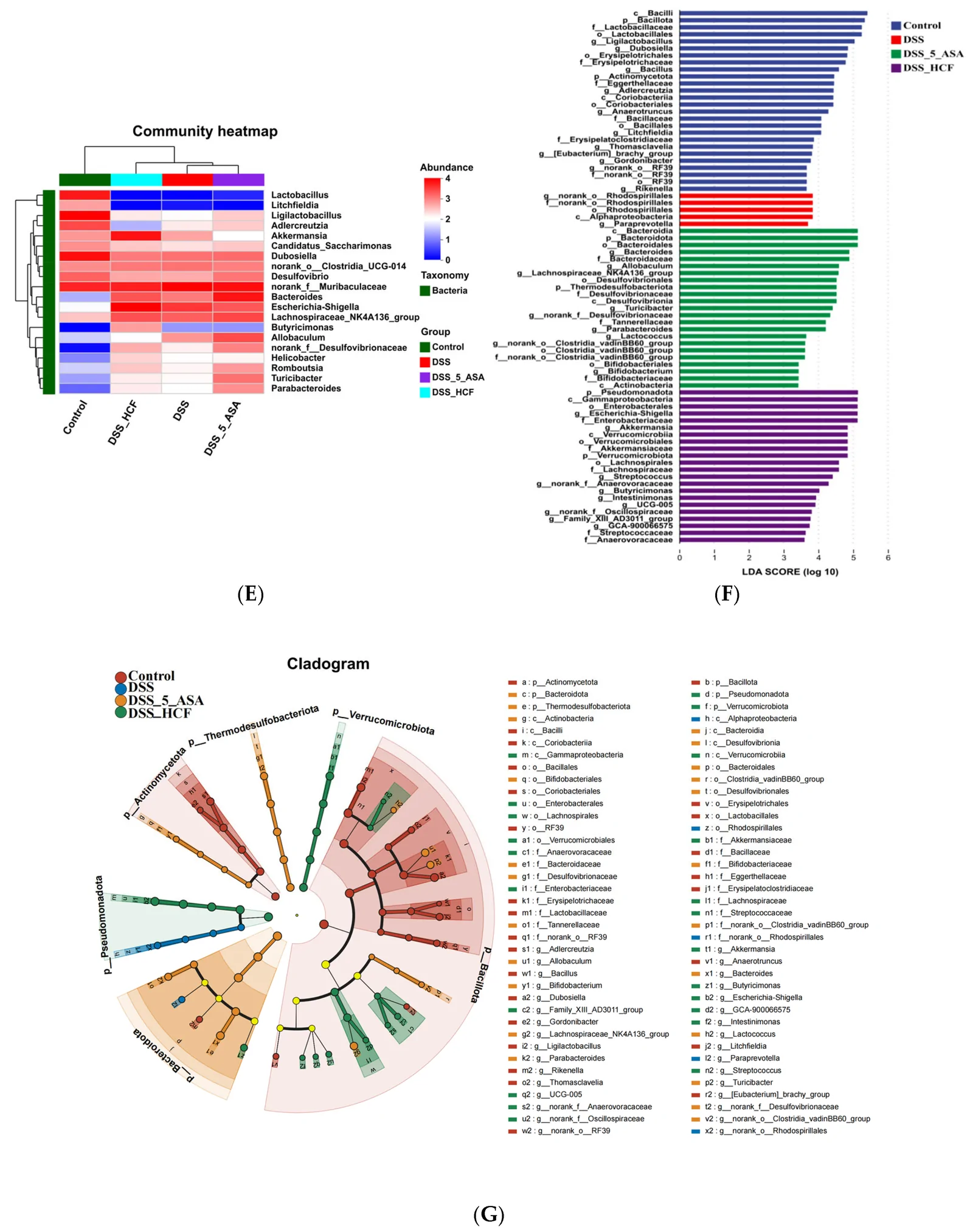
The modulatory effect of EgHCF on the gut microbiota in DSS-induced UC mice. (**A**) Venn diagram. (**B**,**C**) Relative abundance profiles of gut microbiota at the phylum and genus levels. (**D**) Comparison of the Firmicutes/Bacteroidetes ratio among the different experimental groups at the phylum level. (**E**) Community heatmap analysis on the genus level. (**F**) Distribution histogram of differential bacteria based on LDA score (LDA > 4.0; p, c, o, f, g indicate taxonomic boundaries of microorganisms corresponding to phylum, class, order, family, and genus, respectively). (**G**) Cladogram of enriched microbiota generated from LEfSe analysis. Data are shown as mean ± SD (*n* = 5). *^###^ p* < 0.001 versus the control group; * *p* < 0.05 versus the DSS group.

The Venn diagram illustrated the distribution of both shared and unique operational taxonomic units (OTUs) within the gut microbiota across four experimental groups. A total of 763 OTUs were common to all groups, whereas 1070, 469, 1063, and 690 OTUs were uniquely present in the control, DSS, DSS+5-ASA, and DSS+EgHCF groups, respectively (Figure 6A). These results indicate that the gut microbial communities of the different experimental groups exhibited both shared and unique compositional features.

At the phylum level, community abundance analysis showed that *Firmicutes* and *Bacteroidota* remained the dominant phyla across all experimental groups. Compared with the controls, DSS exposure significantly increased the relative abundance of *Bacteroidota* and *Pseudomonadota*, while correspondingly reducing that of *Firmicutes*. Notably, treatment with EgHCF effectively reversed these DSS-induced alterations, as evidenced by a significant increase in *Firmicutes* and a concomitant decrease in *Bacteroidota*. Furthermore, EgHCF treatment significantly enriched the abundance of *Verrucomicrobiota* (Figure 6B,C). At the genus level, the dominant genera differed among the four groups. Compared with the control group, DSS increased the relative abundances of *norank_f_Muribaculaceae* and *Escherichia-Shigella*, while concurrently decreasing those of *Dubosiella* and *Ligilactobacillus*. In contrast, EgHCF treatment markedly reduced *norank_f_Muribaculaceae* while increasing the abundance of *Akkermansia* (Figure 6D). Consistent with these findings, the heatmap analysis revealed that the control group exhibited higher abundances of *Dubosiella*, *Ligilactobacillus*, and *Lactobacillus*, whereas *Escherichia-Shigella* dominated the microbiota of DSS-induced mice. Following EgHCF treatment, *Akkermansia* abundance was significantly increased (Figure 6E). These results indicate that EgHCF modulates the composition and structure of the colonic microbiota in DSS-induced UC mice.

Linear discriminant analysis effect size (LEfSe) analysis was performed to identify the bacterial taxa that were differentially enriched in the gut microbiota of DSS-induced UC mice following EgHCF treatment. In these mice, *Paraprevotella* (*Pseudomonadota*) emerged as the predominant taxon, whereas *Lactobacillus salivarius* (*Firmicutes*) was most abundant in the control group. Treatment with 5-ASA restored *Bacteroidota* as the dominant phylum, accompanied by increased abundances of *Allobaculum*, *Turicibacter*, *Bacteroides*, and *Lachnospiraceae*. In contrast, EgHCF treatment not only restored the DSS-reduced abundance of *Firmicutes* but also enriched *Akkermansia* and *Enterobacteriaceae* (Figure 6F,G).

### 2.7. EgHCF Modulates Gut Microbial Metabolites in DSS-Induced UC Mice

**Figure 7:**
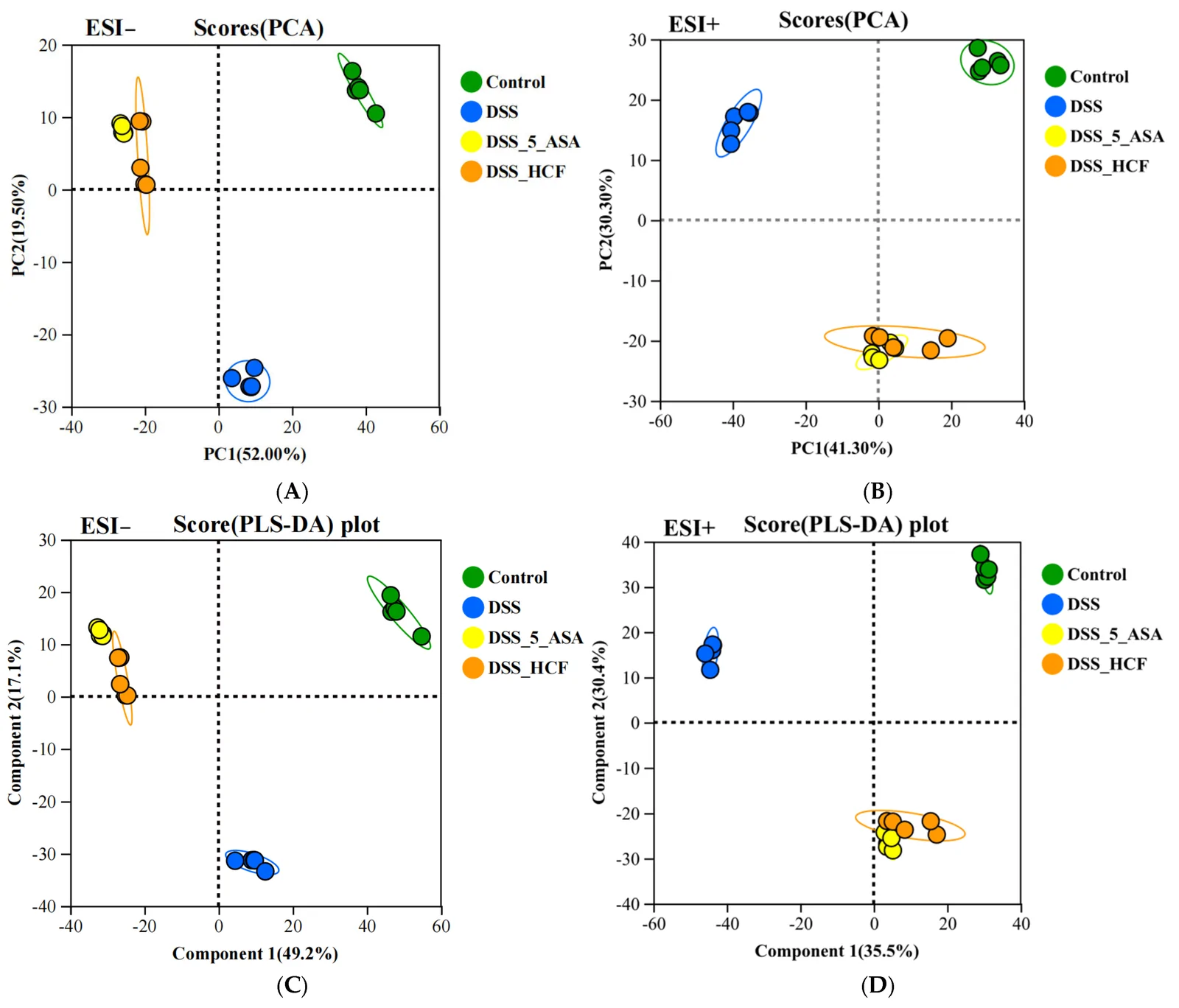

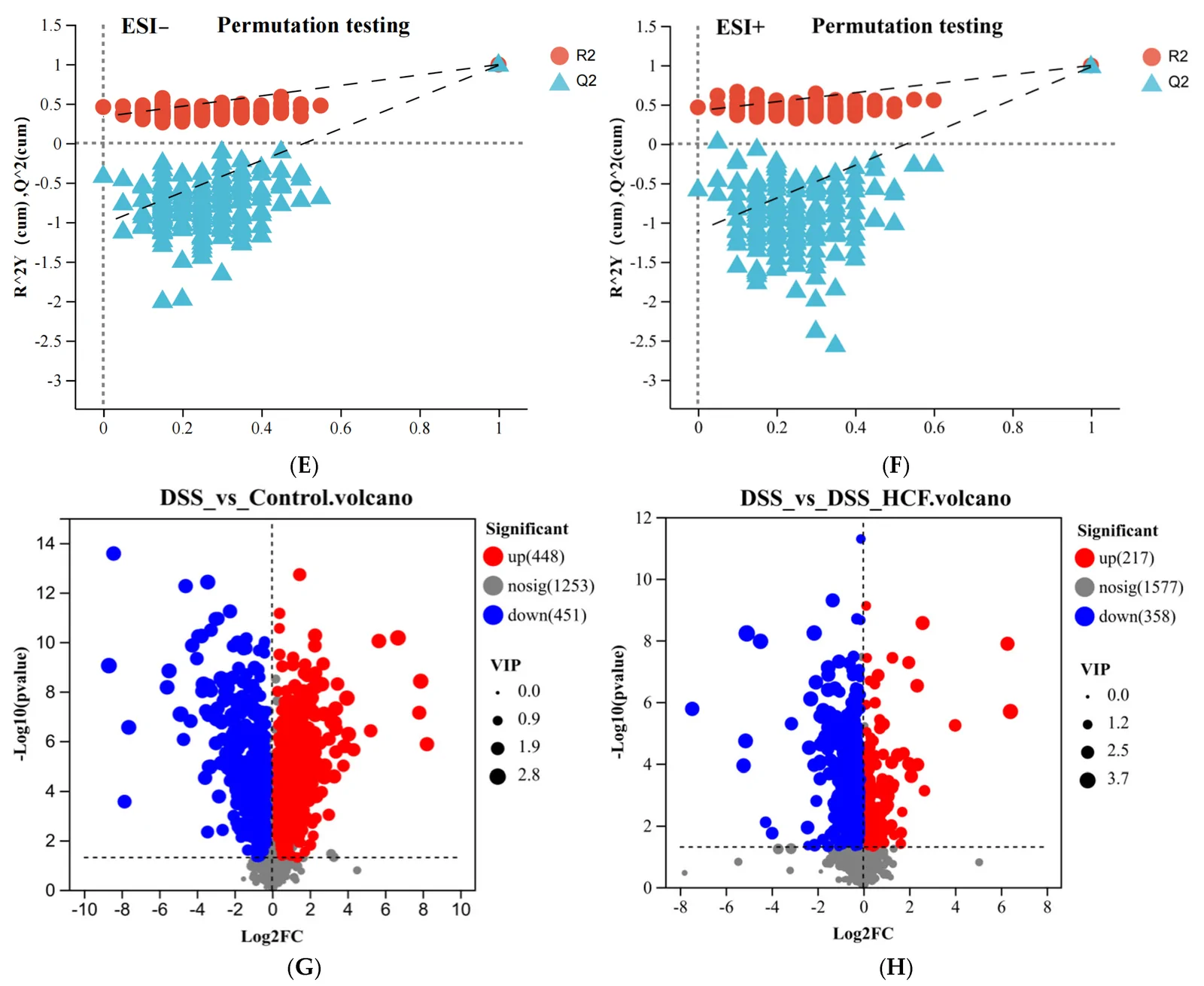
The effect of EgHCF on the comparative analysis of gut microbial metabolites in DSS-induced UC mice. PCA score plot in the negative (**A**) and positive (**B**) mode. PLS−DA score plot in the negative (**C**) and positive (**D**) mode. Negative (**E**) and positive (**F**) ion PLS−DA alignment check. (**G**) The differential metabolites between the DSS and control groups were detected by differential Volcano plots. (**H**) The differential metabolites between the DSS and the DSS+HCF groups were detected by differential Volcano plots. (*n* = 5 per group).

The metabolites produced by gut microbiota serve as key mediators mediating communication between the host and the gut microbiome [17]. To investigate the effects of EgHCF on these interactions, we conducted untargeted metabolomics to assess alterations in gut microbial metabolism in DSS-induced UC mice. PCA revealed distinct cluster separations among the four experimental groups under both positive and negative ion modes (Figure 7A,B). Partial least squares discriminant analysis (PLS-DA) models constructed from ion modes exhibited tight clustering of samples, confirming high data reliability across the four experiment groups, which were clearly separated from one another (Figure 7C,D). Permutation tests (*n* = 200) further validated the stability of the PLS-DA models, ruling out the possibility of overfitting (Figure 7E,F). Volcano plots were subsequently generated to identify differentially expressed metabolites between groups. A total of 899 significantly altered metabolites were detected in the DSS group compared with the control group, among which 448 and 451 were up- and downregulated, respectively (Figure 7G). In contrast, HCF intervention in DSS-treated mice resulted in 217 upregulated and 358 downregulated metabolites relative to the DSS group alone (Figure 7H). In summary, these findings demonstrate that EgHCF treatment significantly modulated the fecal metabolome in DSS-induced UC mice.

**Figure 8:**
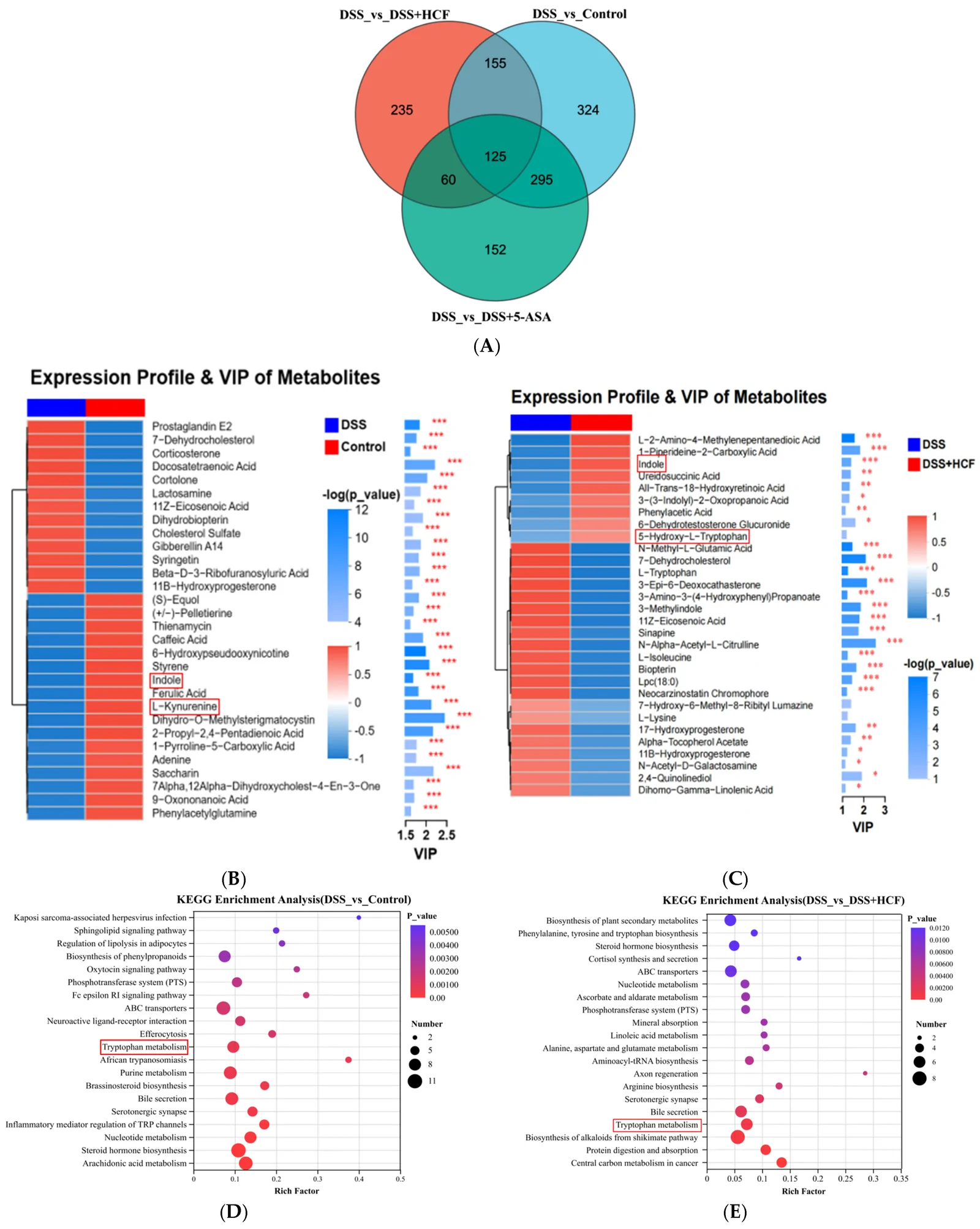

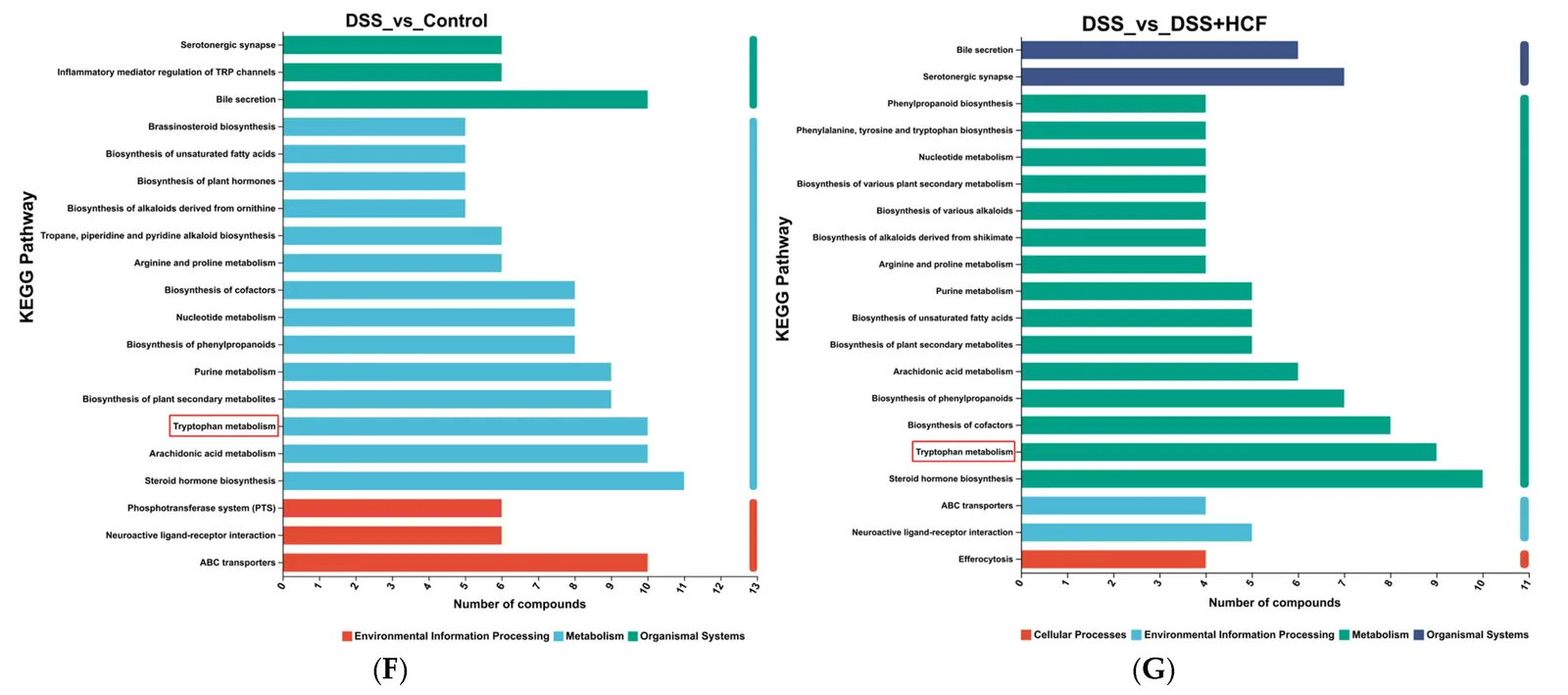
EgHCF modulates gut microbial metabolites in DSS-induced UC mice. (**A**) Venn diagram. (**B**,**C**) VIP bar charts: DSS versus control (**B**) and DSS versus DSS+HCF (**C**). (**D**,**E**) KEGG enrichment analysis. (**F**,**G**) KEGG pathway analysis. (*n* = 5 per group). * *p* < 0.05, ** *p* < 0.01, and *** *p* < 0.001.

Further analyses of fecal metabolite composition across four groups were performed. Differential metabolites that were shared among or unique to each group were compared using Venn diagrams, which identified a total of 125 shared metabolites (Figure 8A). Variable importance in projection (VIP) bar charts were employed to visualize the differential metabolites and their expression trends. The results indicated that tryptophan metabolites played a prominent role in the differential metabolite analysis; specifically, the levels of indole and L-Kynurenic were significantly decreased in the DSS-treated group, while the levels of indole and 5-Hydroxy-L-Tryptophan were markedly increased following EgHCF treatment (Figure 8B,C). The identified metabolites were subsequently subjected to KEGG pathway classification for enrichment and functional analyses. Tryptophan metabolism was found to be the pathway with the highest enrichment rate and also occupied a crucial position in the metabolite functional analysis (Figure 8D–G). Collectively, these findings suggest that the tryptophan metabolism pathway may be a critical metabolic pathway in EgHCF-treated UC mice, with indole serving as the key differential metabolite.

### 2.8. Correlation Analysis of Gut Microbiota and Metabolites

**Figure 9:**
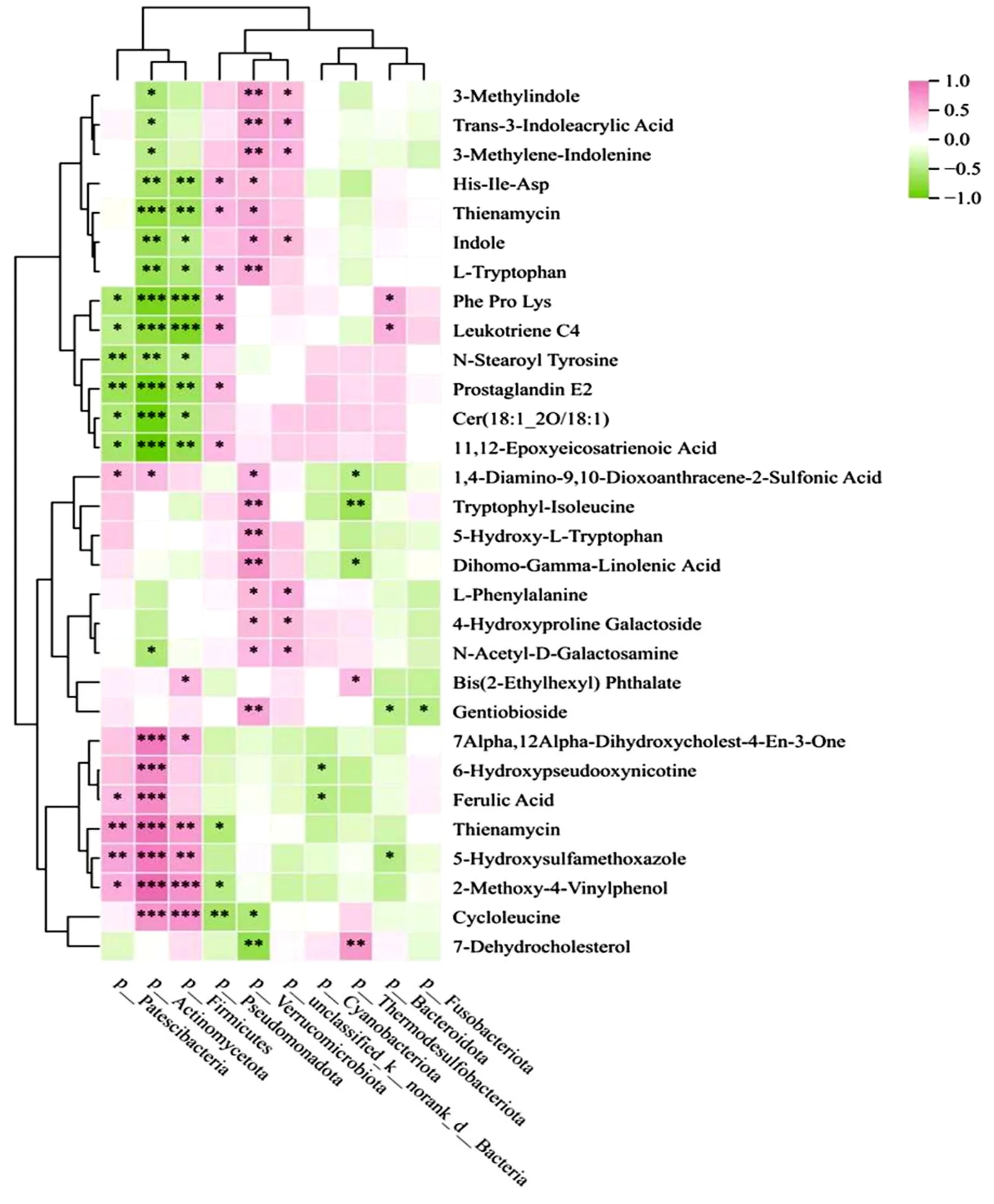
Heatmap of the correlation of the relative abundance of gut microbiota and metabolites in DSS-induced UC mice. The pink and green color represent positive and negative correlation, respectively. * *p* < 0.05, ** *p* < 0.01, and *** *p* < 0.001.

To explore the relationships between phylum-level gut microbiota and host metabolites in DSS-induced UC mice, Spearman correlation analysis was performed. As illustrated in Figure 9, *Actinobacteriota* was significantly negatively correlated with tryptophan-derived metabolites, including trans-3-indoleacrylic acid, L-tryptophan, and indole, whereas these metabolites correlated positively with *Verrucomicrobiota*. Meanwhile, both L-tryptophan and indole exhibited significant negative correlations with *Firmicutes*. Furthermore, *Verrucomicrobiota* and *Firmicutes* were both significantly negatively correlated with leukotriene C4, prostaglandin E2, and 11,12-epoxyeicosatrienoic acid, which are involved in arachidonic acid metabolism.

### 2.9. EgHCF Ameliorates DSS-Induced Barrier Dysfunction in NCM460 Cells

**Figure 10:**
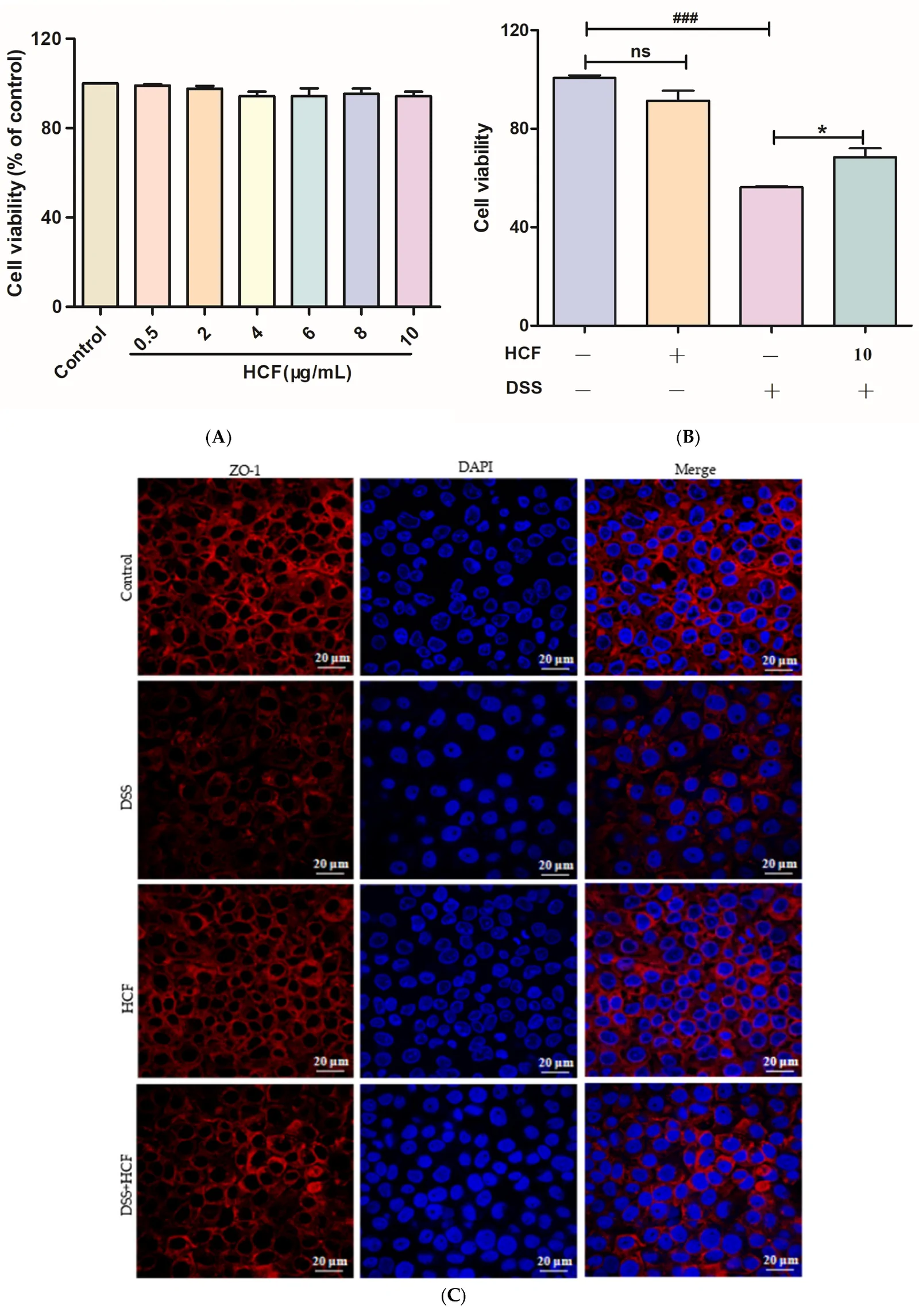

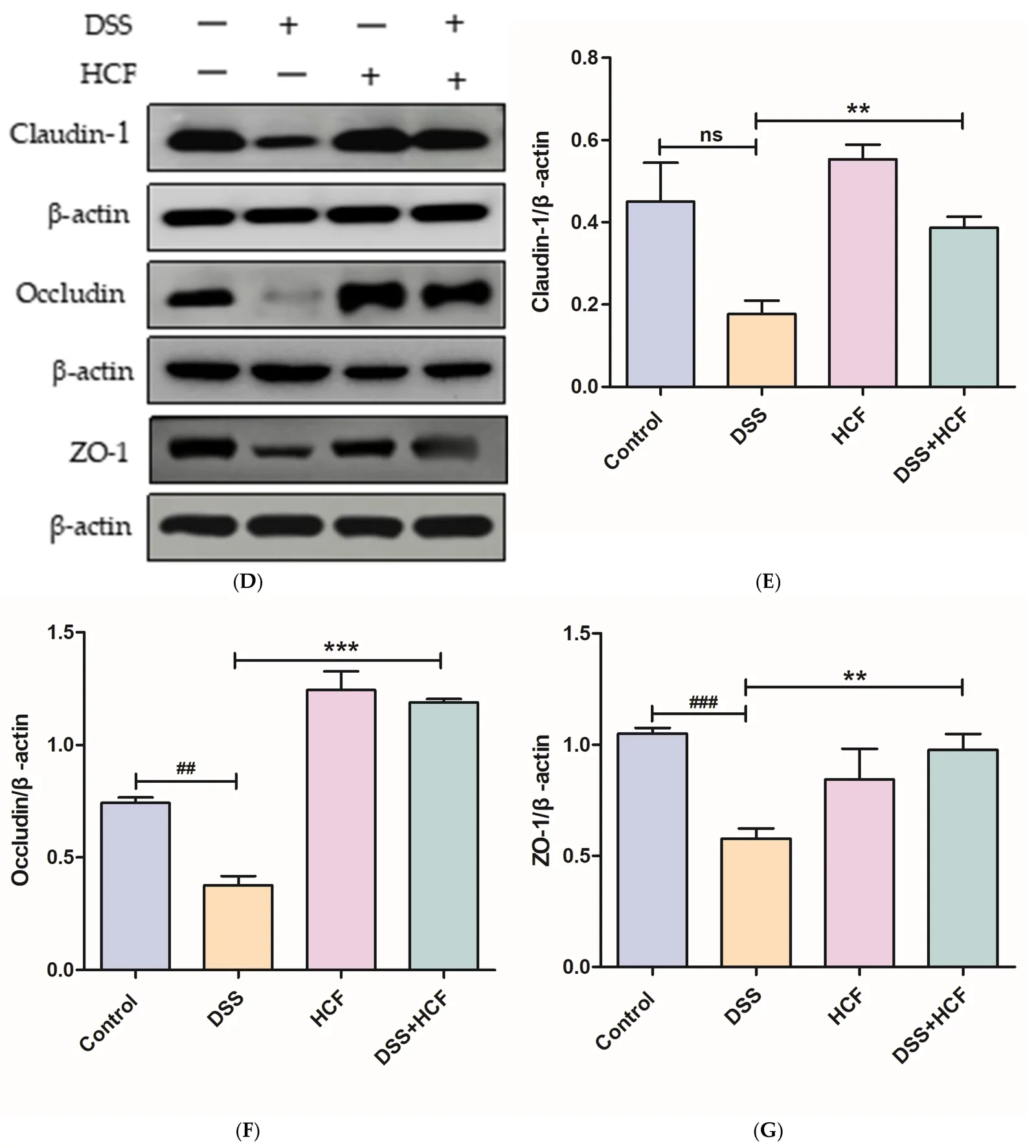
EgHCF ameliorated DSS-triggered barrier damage in NCM460 cells. (**A**) The effect of different concentrations of EgHCF on the viability of NCM460 cells. (**B**) Effect of EgHCF on the viability of DSS-induced NCM460 cells by CCK-8 assay. (**C**) Immunofluorescence analysis of the effect of EgHCF treatment on ZO-1 (red) expression in DSS-induced NCM460 cells. DAPI staining (blue) was used for nuclear counterstaining. (**D**) Western blot analysis of tight junctions protein (Claudin-1, occludin, ZO-1) expression in DSS-induced NCM460 cells treated with EgHCF. (**E**–**G**) Quantification analysis was carried out for the corresponding protein bands. β-actin was used as the loading control. The data are presented as mean ± SD from three independent replicates. *^##^ p* < 0.01, *^###^ p* < 0.001 versus the control group; * *p* < 0.05, ** *p* < 0.01, *** *p* < 0.001 versus the DSS group. “ns” represents no significant difference.

To establish an in vitro model of UC, NCM460 cells were exposed to the inflammatory inducer DSS. The cell counting kit-8 (CCK-8) assay was first performed to evaluate the effect of EgHCF on cell viability following DSS exposure. As shown in Figure 10A, treatment with EgHCF alone (0.5–10 μg/mL) did not significantly affect the viability of NCM460 cells; in contrast, DSS exposure significantly suppressed cell viability compared with the control group, while EgHCF markedly restored cell viability in DSS-treated cells (Figure 10B). Subsequently, the effect of EgHCF on DSS-induced TJ protein expression was examined. IF staining revealed that ZO-1 in the control group was predominantly localized at intercellular junctions, exhibiting smooth contours and a characteristic network pattern, which confirmed that NCM460 cells had formed a stable and dense epithelial monolayer. Conversely, exposure to DSS severely disrupted the continuous network structure of ZO-1; however, co-incubation with EgHCF substantially attenuated this DSS-induced barrier disruption (Figure 10C). Western blot analysis further demonstrated that, compared with the control group, DSS exposure significantly decreased the expression levels of Claudin-1, occludin, and ZO-1, whereas EgHCF significantly restored their expression in DSS-induced NCM460 cells (Figure 10D–G). These results indicate that EgHCF preserves epithelial barrier integrity in NCM460 cells under DSS-induced injury in vitro.

### 2.10. Regulation of Oxidative Stress and Inflammatory Responses by EgHCF in NCM460 Cells

**Figure 11:**
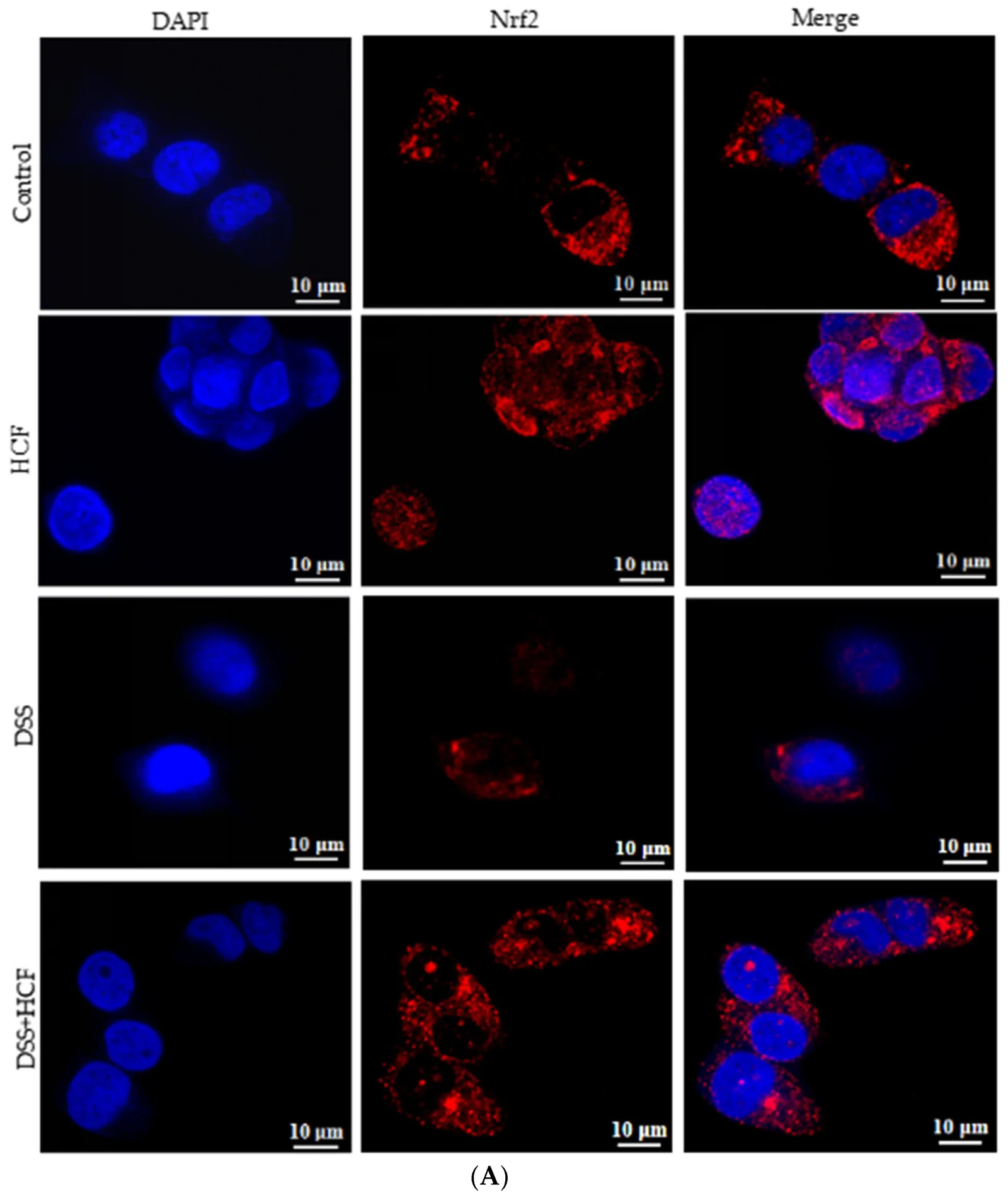

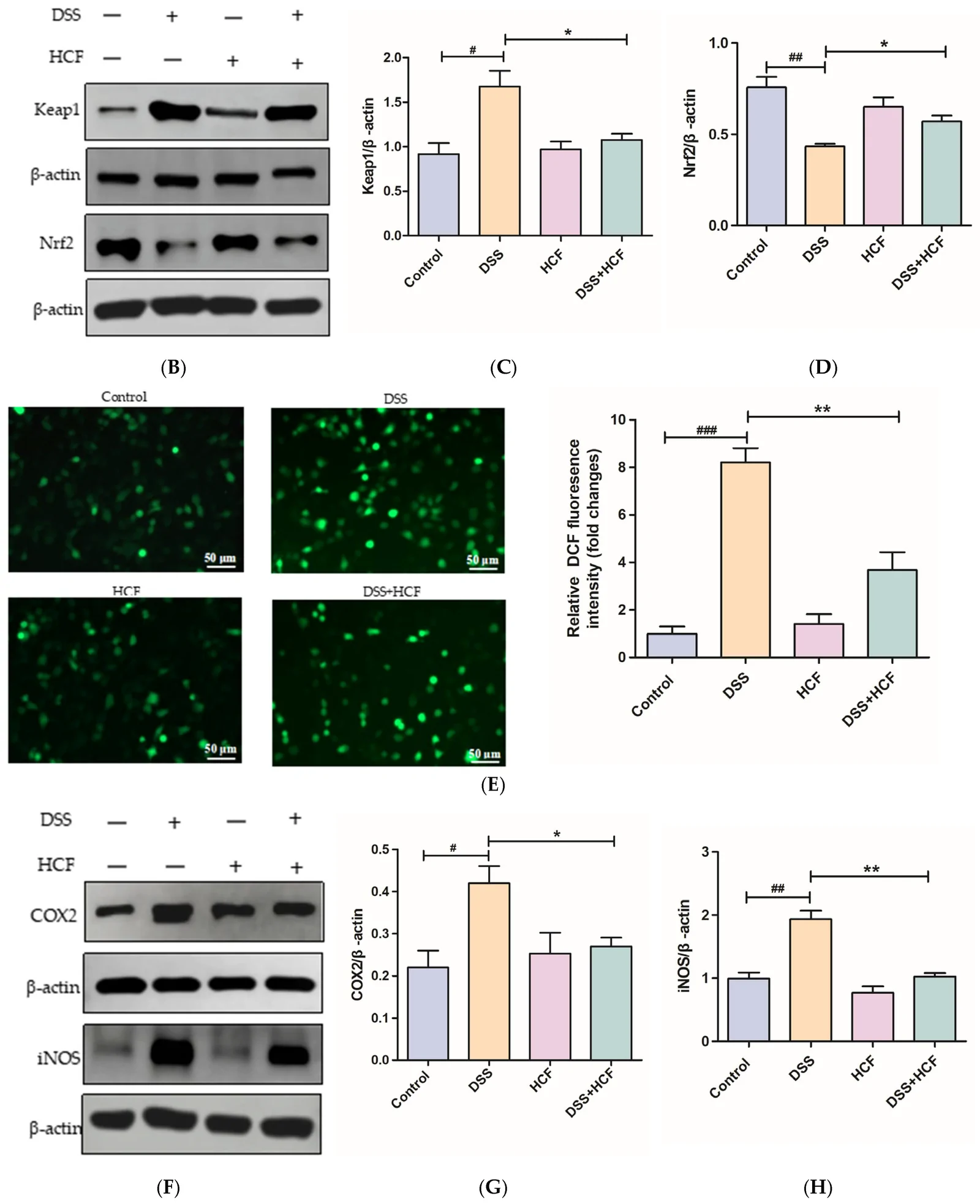
The regulatory effects of EgHCF on oxidative stress and inflammatory responses in NCM460 cells. (**A**) NCM460 cells were pretreated with 2% DSS for 4 h and then stimulated with EgHCF for 24 h. Immunofluorescence staining was performed to detect Nrf2 (red), with nuclei counterstained using DAPI (blue). (**B**) Representative Western blot analysis of Keap1 and Nrf2 protein expression levels in NCM460 cells. (**C**,**D**) Quantitative analysis of Keap1 and Nrf2 protein levels normalized to β-actin. (**E**) Intracellular ROS level detection using a DCFH-DA probe (green fluorescence) analyzed by fluorescence microscopy. (**F**) Western blot analysis of the expression levels of COX2 and iNOS proteins. (**G**,**H**) Quantitative analysis of COX2 and iNOS protein levels normalized to β-actin. Results are mean ± SD and represent one of three independent experiments. The data are presented as mean ± SD from three independent replicates. *^#^ p* < 0.05, *^##^ p* < 0.01, *^###^ p* < 0.001 versus the control group; * *p* < 0.05 and ** *p* < 0.01 versus the DSS group.

To investigate the regulation effect of EgHCF on the Keap1/Nrf2 signaling pathway in DSS-induced NCM460 cells, we examined Nrf2 subcellular localization using IF staining. The results showed that DSS treatment not only downregulated Nrf2 expression but also suppressed its nuclear translocation, and this effect was substantially restored upon EgHCF intervention (Figure 11A). Consistent with this observation, Western blot analysis showed that the protein expression level of Keap1 was significantly elevated in DSS-treated cells compared with the control group, while EgHCF treatment markedly attenuated this upregulation. In contrast, the protein expression of Nrf2 was significantly downregulated in DSS-induced NCM460 cells, and this reduction was largely restored following EgHCF treatment (Figure 11B–D). Furthermore, the 2′,7′-dichlorodihydrofluorescein diacetate (DCFH-DA) fluorescence assay demonstrated that reactive oxygen species (ROS) levels were markedly elevated in DSS-stimulated NCM460 cells relative to the control group; however, EgHCF intervention significantly attenuated this fluorescence intensity (Figure 11E). In parallel, DSS treatment significantly upregulated the expression levels of COX2 and iNOS in NCM460 cells compared to the control group, while EgHCF intervention markedly suppressed the DSS-induced elevation of these inflammatory proteins (Figure 11F–H).

## 3. Discussion

Helminth-derived proteins possess the capacity to modulate the host immune system by regulating the production of inflammatory cytokines both locally and systemically. Furthermore, they can alleviate the severity of inflammatory diseases by enhancing regulatory T-cell function, thus emerging as a highly promising strategy for mitigating inflammation in UC [18]. Previous studies have demonstrated that HCF and the laminated layer derived from *E. granulosus* enhance protection against colitis in mice by increasing anti-inflammatory and antioxidant properties [11]. EgHCF is a complex mixture of excretory/secretory products derived from PSCs and the GL, comprising glycolipids, proteins, carbohydrates, and salts, and incorporates both host-derived molecules and parasite macromolecules. Importantly, it harbors the major immunodiagnostic protein antigens, which confer distinctive antigenic properties upon the parasite [19,20].

EgHCF has previously been shown to exert a significant therapeutic effect against sepsis in mice by upregulating pro-inflammatory factors, thereby improving survival rates and reducing tissue damage [21]. Building on these findings, we evaluated the regulatory effects of EgHCF in DSS-induced UC mice. EgHCF treatment alleviated clinical symptoms and restored colonic integrity, demonstrating its protective effects against DSS-induced colitis. In addition, EgHCF inhibited Th1 pro-inflammatory cytokines (IL-1β and IL-6) while increasing the Th2 cytokine IL-10, and attenuated iNOS and COX2 expression in colonic tissues. These results are consistent with those of Lin et al., who showed that EgCF reduced TNF-α, IL-12, and IL-6 production while increasing IL-10 in LPS-stimulated peritoneal macrophages [22]. This immunomodulation effect is likely mediated through regulatory T-cell activation, IL-10 involvement, and a shift toward Th2 polarization accompanied by suppression of Th1 responses [18]. These findings suggest that EgHCF may exert its protective effects in UC by orchestrating a coordinated shift toward an anti-inflammatory immune profile.

During UC onset, activated immune cells and intestinal microorganisms generate ROS that overwhelm endogenous scavenging capacity, leading to ROS accumulation [23,24]. Our results showed that EgHCF suppressed DSS-induced ROS production in NCM460 cells; consistently, in DSS-induced UC mice, EgHCF treatment significantly restored antioxidant markers, as evidenced by enhanced levels of GSH, T-AOC, and SOD activity. Given that excessive ROS induce oxidative stress and DNA damage through an imbalance between antioxidant defenses and elevated ROS/reactive nitrogen species (RNS) production [25], the restoration of these antioxidant markers by EgHCF is closely associated with its capacity to scavenge surplus ROS. In additional, the Keap1/Nrf2 signaling pathway is one of the most important defense systems against oxidative stress in UC pathogenesis [26]. In our study, DSS suppressed Nrf2 expression while elevating Keap1 levels; in contrast, EgHCF treatment reversed these alterations, upregulated the mRNA levels of Nrf2 downstream targets, and promoted Nrf2 nuclear translocation in NCM460 cells. Given the key role of the Keap1/Nrf2 signaling pathway in regulating the antioxidant response, the activation of this pathway by EgHCF may contribute substantially to its protective effects against DSS-induced colitis. Nevertheless, whether this signaling pathway indeed mediates the antioxidant and anti-inflammatory actions of EgHCF remains to be definitively established. Therefore, further studies are required using Nrf2 knockdown or specific pathway inhibitors (e.g., ML385) to establish a causal relationship between EgHCF-induced Nrf2 signalling and its beneficial effects on UC.

The association between UC and the gut microbiome has attracted considerable attention [17,27]. The gut microbiota helps maintain intestinal epithelial barrier integrity, which is not a static physical shield but a dynamic interface interacting extensively with microbes and immune cells [28,29]. To determine whether EgHCF treatment alters the gut microbial community, we performed 16S rRNA gene sequencing on fecal samples from UC mice. The results revealed that EgHCF treatment significantly enhanced both the α- and β-diversity of the gut microbiota, the former reflecting the diversity within a single microbial community and the latter assessing the similarity or dissimilarity of microbial communities across different samples [30]. In addition, the DSS-induced colitis model is associated with alterations across multiple microbial phyla, including *Firmicutes*, *Bacteroidetes*, *Verrucomicrobia*, and *Proteobacteria*; among these, accumulating evidence has highlighted that *Firmicutes* and *Bacteroidetes* play a particularly critical role in the pathogenesis of UC [31,32]. In our study, *Firmicutes* and *Bacteroidota* dominated the microbiota of all groups. DSS exposure significantly elevated the abundances of *Bacteroidota* and *Pseudomonadota* while reducing that of *Firmicutes*; subsequent EgHCF treatment reversed these shifts and markedly increased *Verrucomicrobiota*. At the genus level, DSS markedly elevated the relative abundance of *norank_f_Muribaculaceae*, while EgHCF treatment not only attenuated this increase but also promoted the abundance of *Akkermansia*, which plays a pivotal role in maintaining intestinal barrier integrity and modulating immune responses, thereby limiting the onset of chronic inflammation [33]. Conversely, the abundance of *norank_f_Muribaculaceae* has been reported to be negatively correlated with inflammatory markers [34]. Taken together, our findings indicate that EgHCF treatment alleviated DSS-induced intestinal microbial dysbiosis in UC mice; however, whether this microbial restoration directly drives the attenuation of inflammation, or is instead a consequence of it, requires further investigation.

Gut microbiota-derived metabolites are structurally diverse and play a crucial role in organismal signaling and immune homeostasis, serving as essential intermediaries in host and microbe communication [35]. In the present study, untargeted metabolomic profiling of fecal samples from EgHCF-treated mice with DSS-induced UC identified 125 shared gut metabolites, with tryptophan metabolism emerging as the predominant pathway. Tryptophan, an essential amino acid, is metabolized via three principal pathways—the indole, kynurenine, and serotonin pathways [36]—and its metabolism is well recognized for modulating host immunity and sustaining microbial equilibrium [37]. Among these, indole derivatives function as endogenous AhR ligands that regulate inflammation by inhibiting the NF-κB and NLRP3 pathways and by modulating immune cell polarization and T-cell differentiation [38]. Notably, indole levels, which were significantly reduced by DSS treatment, were markedly restored by EgHCF treatment. Given that indole derivatives derived from bacterial tryptophan metabolism reinforce the intestinal barrier through upregulation of tight junction proteins and induction of anti-inflammatory cytokines [39], this restoration of intestinal indole levels may represent an important contributor to the protective efficacy of EgHCF against DSS-induced UC; however, this possibility warrants further experimental validation. Furthermore, correlation analysis revealed positive associations between *Verrucomicrobiota* and tryptophan metabolites, including trans-3-indoleacrylic acid, L-tryptophan, and indole, linking the observed microbial shifts to the concurrent metabolic changes. These findings suggest that EgHCF may exert a multifaceted protective effect by modulating gut microbiota composition and host tryptophan metabolism. Nevertheless, additional experiments are required to validate these metabolite changes and to elucidate the underlying mechanistic pathways.

In summary, EgHCF treatment effectively alleviated the symptoms of DSS-induced UC in mice, while preserving intestinal barrier integrity and exerting both anti-inflammatory and antioxidant effects. Moreover, EgHCF enhanced gut microbial diversity and ameliorated DSS-induced gut dysbiosis, notably increasing the relative abundance of the *Firmicutes* and *Verrucomicrobia* phyla, as well as modulated the tryptophan metabolic pathway. These findings suggest that EgHCF may play a protective role against DSS-induced colitis. Nevertheless, the present results remain exploratory, as they were obtained in a DSS-induced acute model and an in vitro epithelial cell, with limited sample sizes for several key analyses. In addition, sterility testing confirmed that the EgHCF preparation was free of detectable bacterial contamination, but potential contamination with other pathogens (e.g., mycoplasma and fungi) cannot be completely excluded. Therefore, a comprehensive characterization of EgHCF, together with further in-depth investigations, is warranted to determine whether its exerts long-term protection against ulcerative colitis.

## 4. Materials and Methods

### 4.1. Chemicals and Reagents

Dextran sulfate sodium (DSS, MW; 36–50 kDa) was purchased from MP Biomedicals (San Diego, CA, USA). RIPA lysis buffer, 3,3′-diaminobenzidine (DAB) chromogen, the bicinchoninic acid (BCA) assay kit, and the MPO activity assay kit were obtained from Solarbio life sciences (Beijing, China). ELISA kits (IL-10, IL-1β, IL-6) were purchased from Lianke Biotechnology (Hangzhou, China). Reduced glutathione (GSH), superoxide dismutase (SOD), and total antioxidant capacity (T-AOC) assay kits were obtained from Nanjing Jiancheng Bioengineering Institute (Nanjing, China). The alanine aminotransferase (ALT/GPT) activity (E-BC-K235-M), aspartate aminotransferase (AST/GOT) activity (E-BC-K236-M), urea colorimetric (E-BC-K183-M), and Cr colorimetric (E-BC-K188-M) assay kits were purchased from Elabscience Biotechnology Co., Ltd. (Wuhan, China).

The following antibodies were used in this study: rabbit anti-occludin (27260-1-AP), rabbit anti-Claudin-1 (28674-1-AP), rabbit anti-ZO-1 (21773-1-AP), and rabbit anti-MPO (22225-1-AP), all purchased from Proteintech (Wuhan, China); rabbit anti-iNOS (YM8628), rabbit anti-COX-2 (YM8171), rabbit anti-Keap1 (YM8283), and rabbit anti-Nrf2 (YM8624), all purchased from Immunoway Biotechnology Company (Suzhou, China); rabbit anti-β-actin antibody (HY-P87821), purchased from MedChemExpress Ltd. (Monmouth Junction, NJ, USA); goat anti-rabbit IgG H&L (Alexa Fluor^®^488, ab150077), purchased from Abcam Ltd. (Cambridge, UK); Cy3-labeled goat anti-rabbit IgG H&L (A0516), purchased from Beyotime Biotechnology (Shanghai, China); and goat anti-rabbit IgG H&L (bs-0295G), purchased from Bioss Biotechnology (Beijing, China).

### 4.2. Preparation of EgHCF

The livers from sheep naturally infected with *E. granulosus* were collected at a slaughterhouse in Urumqi, Xinjiang. Five livers with obvious hydatid cysts were obtained from two independent batches of sheep, with varying cyst numbers per liver. After surface disinfection of the livers, the contents of the hydatid cysts were aspirated and centrifuged at 1000 g for 10 min. The supernatant was then filtered through a 0.22-μm filter. The concentration of the resulting EgHCF was determined using the BCA kit (Beyotime Biotechnology, Shanghai, China) and the two batches of EgHCF exhibited concentrations of 1.56 mg/mL and 1.83 mg/mL, respectively. Then, the EgHCF from all samples was pooled and mixed, and the endotoxin activity was assessed using a ToxinSensorTM Chromogenic LAL Endotoxin Assay Kit (GenScript Biotech Corp, Nanjing, China). The endotoxin level was lower than <1 EU/mL, indicating that its potential impact in the EgHCF is minimal. The protein profile of EgHCF was analyzed by sodium dodecyl sulfate polyacrylamide gel electrophoresis (SDS-PAGE), as shown in Supplementary Figure S1A, EgHCF displayed multiple protein bands. Western blot analysis using serum from EgHCF-immunized mice as the primary antibody revealed multiple immunoreactive bands (Supplementary Figure S1B). Additionally, an antibiotic-free LB solid medium was used to test for bacterial contamination, and no bacteria were detected in the EgHCF (Supplementary Figure S2). The EgHCF was then stored at −80 °C for subsequent use.

### 4.3. Animals and Experimental Protocol

All animals were handled in strict accordance with the Animal Ethics Procedures and Guidelines of the People’s Republic of China, and the experimental protocol was approved by the Animal Ethics Committee of Xinjiang Medical University (IACUC-20240514-40). Male C57BL/6 mice (6–8 weeks, 22 ± 2 g) were purchased from the Experimental Animal Center of Xinjiang Medical University (Urumqi, China) and housed under specific pathogen-free (SPF) conditions (temperature: 25 ± 3 °C; relative humidity: 55–60%; 12 h light/dark cycle) for one week prior to the experiment.

Mice were randomly divided into four groups (*n* = 8 per group)—Control, DSS, DSS+5-ASA (100 mg/kg), and DSS+EgHCF—using a computer-generated sequence produced with IBM SPSS Statistics version 26.0 (IBM Corp., Armonk, NY, USA). The randomization sequence and group allocation codes were generated by an investigator who was not involved in animal handling, outcome assessment, or any subsequent data analyses, thereby minimizing the risk of selection bias. The sample size was not determined by a formal a priori power calculation; instead, the minimum number of animals needed to yield reliable and interpretable statistical data was used, in accordance with the 3R principles (Replacement, Reduction, and Refinement) of animal research [40]. During the experiment, the control group received purified water, while the other groups were provided with 3% (*w*/*v*) DSS solution (dissolved in purified water) ad libitum. In addition, mice in the DSS+5-ASA and DSS+EgHCF groups received daily intraperitoneal injections of the corresponding drugs (total volume 200 μL) for 7 consecutive days. At the end of the treatment period, all mice were anesthetized, the blood samples were collected via retro-orbital puncture, and the animals were subsequently euthanized (Figure 1A). Body weight, fecal consistency, and fecal occult blood were recorded daily for each mouse, and the disease activity index (DAI) scores were calculated according to the scoring system (Table 1) [41]. After euthanasia, spleen weights were measured, and the colons, sera, and feces were collected and stored at −80 °C for further analysis.

### 4.4. Cell Culture

The human colonic epithelial cell line NCM460 was purchased from Procell (Wuhan, China) and cultured in Dulbecco’s Modified Eagle Medium (DMEM; Procell, Wuhan, China) supplemented with 1% antibiotics (100 U/mL penicillin–100 μg/mL streptomycin) and 15% fetal bovine serum (FBS; Gibco, Thermo Fisher Scientific, Waltham, MA, USA). Cells were maintained in a humidified incubator at 37 °C with 5% CO_2_.

### 4.5. CCK-8 Assay

NCM460 cells were seeded in 96-well plates (2 × 10^3^ cells/well). After pre-incubation with or without 2% (*w*/*v*) DSS for 4 h, cells were treated with 0.5–10 μg/mL EgHCF for 24 h. Phosphate-buffered saline (PBS)-only and DSS-only groups served as negative controls. For cell viability assessment, 10 μL of CCK-8 solution (Elabscience Biotechnology Co., Ltd, Wuhan, China) was added to each well and incubated at 37 °C for 2 h. Absorbance was measured at 450 nm using a microplate reader (Thermo Scientific, Waltham, MA, USA). All experiments were independently performed in triplicate.

### 4.6. ROS Assay

NCM460 cells were seeded at a density of 2 × 10^4^ cells per well and incubated with or without 2% DSS for 4 h, followed by treatment with EgHCF (10 μg/mL) for an additional 24 h. Cells treated with DSS alone served as the control group. After incubation, the cells were harvested, resuspended in 2 μM DCFH-DA solution (Solarbio life sciences, Beijing, China), and incubated at 37 °C for 20 min in the dark. Following three washes with PBS, the fluorescence intensity was observed under a fluorescence microscope (Leica, Wetzlar, Germany). All experiments were independently performed three times.

### 4.7. Histopathological Analysis

After the end of the experiment, colons were rapidly excised from the mice, rinsed with sterile saline, and measured for their length. The colon tissues (*n* = 3 per group) were then fixed in 4% paraformaldehyde for 24 h, routinely embedded in paraffin, and sectioned into 5 μm thick slides for subsequent staining [42]. Hematoxylin and eosin (H&E) staining was performed to evaluate histopathological changes, including inflammatory cell infiltration, mucosal edema, crypt destruction, and epithelial cell damage. PAS staining was performed to evaluate the colonic mucus thickness.

### 4.8. IHC Evaluation

IHC staining was performed on paraffin-embedded colon tissue sections (*n* = 3 per group). After deparaffinization and rehydration, the sections were subjected to heat-induced antigen retrieval, followed by blocking of endogenous peroxidase activity and nonspecific binding. The samples were then incubated with a primary antibody against MPO, and subsequently reacted with the corresponding horseradish peroxidase (HRP)-conjugated secondary antibodies. Immunoreactivity was visualized using DAB as the chromogen, and the sections were counterstained with hematoxylin, dehydrated, and mounted. For quantitative evaluation, the percentage of MPO-positive staining area per field was measured using ImageJ software (version 1.52a; National Institutes of Health, Bethesda, MD, USA; https://imagej.net/ij/, accessed on 12 October 2025).

### 4.9. Immunofluorescence Assay

Immunofluorescence (IF) staining was performed on both paraffin-embedded colon tissue sections and NCM460 cells. The tissue sections were baked at 60 °C for 4 h, then deparaffinized, rehydrated, and subjected to heat-induced antigen retrieval in 10 mM sodium citrate buffer. For cell experiments, NCM460 cells were treated with or without 2% DSS for 4 h, followed by exposure to EgHCF for 24 h, and subsequently fixed with 4% paraformaldehyde for 15 min. Both tissue sections and cells were permeabilized with 0.3% Triton X-100 and blocked with 0.5% bovine serum albumin (BSA, Solarbio life sciences, Beijing, China), then incubated with primary antibodies overnight at 4 °C. After washing, the samples were incubated with fluorescent-conjugated secondary antibodies, and nuclei were counterstained with DAPI for 10 min. Images were captured using a laser scanning confocal microscope (Leica, Wetzlar, Germany).

### 4.10. Assessment of Oxidative Stress Markers and MPO Activity

Colon tissues (*n* = 3 per group) were rinsed with pre-cooled saline and homogenized to prepare 10% (*w*/*v*) tissue homogenates. After centrifugation at 5000× *g* for 10 min at 4 °C, the supernatants were collected. The protein concentration was determined using a BCA kit. Levels of reduced GSH, SOD, and T-AOC were measured following the manufacturer’s instructions from the Nanjing Jiancheng Bioengineering Institute (Nanjing, China). MPO activity was assayed according to the manufacturer’s instructions using a commercial kit (Solarbio life sciences, Beijing, China).

### 4.11. ELISA Detection of Cytokine Levels

Serum (*n* = 8 mice per group) levels of inflammatory cytokines, including IL-10, IL-1β, and IL-6, were quantified using commercial enzyme-linked immunosorbent assay (ELISA) kits (Lianke Biotechnology, Hangzhou, China) according to the manufacturer’s protocols.

### 4.12. Serum Biochemical Analysis

The levels of urea, Cr, ALT, and AST in mouse serum were strictly measured according to the instructions of the commercial kits (Elabscience Biotechnology Co., Ltd, Wuhan, China).

### 4.13. qRT-PCR Analysis

Total RNA was extracted from mouse colon tissues (*n* = 3 per group) using Trizol reagent (Invitrogen, Thermo Fisher Scientific, Carlsbad, CA, USA), and RNA concentration and purity were determined using a NanoDrop Spectrophotometer (Thermo Scientific, Wilmington, DE, USA). Subsequently, 2 μg of total RNA was reverse-transcribed into cDNA using a PrimeScript™RT reagent Kit with gDNA Eraser (Takara, Dalian, China) according to the manufacturer’s protocol. The mRNA expression levels of target genes were quantified by qRT-PCR using cDNA as the template. β-actin was used as the endogenous reference gene. Relative gene expression levels were calculated using the 2^−ΔΔCt^ method [43]. All primer sequences are listed in Table 2.

### 4.14. Western Blot Analysis

Total protein was extracted from mice colon tissues or cells using RIPA lysis buffer, and protein concentration was measured using a BCA kit. Subsequently, protein samples (20 μg/lane) were separated by SDS-PAGE and transferred onto PVDF membranes (Millipore, Billerica, MA, USA). The membranes were blocked with 5% skim milk in PBST (pH 7.4) for 2 h at 37 °C, followed by overnight incubation with primary antibodies at 4 °C. After washing, the membranes were incubated with HRP-conjugated secondary antibodies for 1 h at room temperature. Protein bands were visualized using enhanced chemiluminescence (Biosharp, Hefei, China), and quantification analysis was performed with ImageJ software.

### 4.15. Gut Microbiota Analysis by 16S rRNA Sequencing

Fecal samples collected from mice were immediately snap-frozen in liquid nitrogen and stored at −80 °C until further use. Genomic DNA was extracted from fecal samples (*n* = 5), and its integrity was evaluated by agarose gel electrophoresis, while its concentration and purity (A260/A280 ratio) were determined with a NanoDrop spectrophotometer. For 16S rRNA sequencing, genomic DNA was used as a template for PCR amplification of the V3-V4 region using specific primers 338F (5′-ACTCCTACGGGAGGCAGCAG-3′) and 806R (5′-GGACTACHVGGGTWTCT AAT-3′). Library construction and sequencing were conducted by Majorbio Technology Co., Ltd. (Shanghai, China).

Statistical analyses were performed using R software (version 4.3.1; R Foundation for Statistical Computing, Vienna, Austria; https://www.R-project.org/) and the Majorbio cloud platform (www.majorbio.com, accessed on 9 January 2026). Sequences were clustered into Amplicon Sequence Variants (ASVs) based on sequence similarity. A sampling depth, defined as the number of sequences in the sample with the fewest sequences, was used to rarefy the ASV abundance table and standardize comparisons across samples.

α-diversity metrics, including Observed ASVs and Chao1, Shannon, and Simpson indices, were computed, and differences among groups were compared using one-way ANOVA followed by Tukey’s post hoc test, or the Kruskal–Wallis test followed by Dunn’s post hoc test, depending on data distribution (assessed via the Shapiro–Wilk test). For pre-and post-colitis comparisons, unpaired *t*-tests or Mann–Whitney U tests were applied, as appropriate.

β-diversity was assessed using PCA, PCoA, NMDS, and NCM based on Bray–Curtis dissimilarity. Differences in β-diversity among groups were further evaluated by permutation-based multivariate analysis of variance (PERMANOVA/Adonis), with statistical significance set at *p* < 0.05. Microbiome composition data were obtained by normalizing to the total number of reads in each sample (relative abundance).

Microbiome composition data at the phylum and genus levels were obtained by normalizing to the relative abundance (proportion of total reads per sample). Differential abundance analysis was performed using Analysis of Compositions of Microbiomes with Bias Correction (ANCOM-BC), with statistical significance set at *p* < 0.05. To identify significantly important microbial taxa as potential biomarkers, LEfSe analysis was performed with a default LDA score threshold of log_10_ (LDA) > 4.0.

### 4.16. Untargeted Metabolomics

Fecal samples (20 ± 5 mg, *n* = 5 per group) from each group were transferred into 2 mL centrifuge tubes containing a grinding bead with a diameter of 6 mm. Metabolites were extracted using 400 μL of extraction solution (methanol:water = 4:1, *v*/*v*) supplemented with 0.02 mg/mL L-2-chlorophenylalanine. The samples were ground in a cryogenic tissue grinder for 6 min (50 Hz, −10 °C), followed by cryogenic ultrasonic extraction for 30 min (40 kHz, 5 °C). Subsequently, the sample solutions were incubated at −20 °C for 30 min and centrifuged at 13,000× *g* for 15 min at 4 °C; then the supernatants were collected and transferred into LC-MS vials for analysis. Chromatographic separation was performed on an HSS T3 column (100 mm × 2.1 mm i.d., 1.8 μm; Waters, Milford, MA, USA). Mobile phase A consisted of water/acetonitrile (95:5, *v*/*v*) containing 0.1% formic acid, while mobile phase B comprised acetonitrile/isopropanol/water (47.5:47.5:5, *v*/*v*/*v*) containing 0.1% formic acid.

Raw untargeted metabolomics data were processed using Progenesis QI (Waters Corporation, Milford, MA, USA) for alignment, peak detection, and peak area integration. Metabolite identification was performed by searching the Human Metabolome Database (HMDB, version 5.0, University of Alberta, Edmonton, AB, Canada; http://www.hmdb.ca/, accessed on 13 January 2026), METLIN (Scripps Research Institute, La Jolla, CA, USA; https://metlin.scripps.edu/, accessed on 17 January 2026), and the Majorbio in-house database. To minimize experimental and analytical bias, features with more than 20% missing values in any group were removed; the remaining missing values were imputed using the minimum value detected across all samples, and the data were then normalized by total sum. Variables with a relative standard deviation (RSD) exceeding 30% in quality control (QC) samples were further excluded, followed by log10 transformation to generate the final data matrix.

PCA and PLS-DA were performed under both positive and negative ion modes to visualize the overall sample distribution and inter-group separation. Orthogonal partial least squares discriminant analysis (OPLS-DA) was further conducted to maximize group separation and to calculate the VIP scores. The robustness and validity of the OPLS-DA models were assessed by permutation testing (200 permutations), with the R2Y and Q2Y intercepts reported. For univariate analysis, Welch’s *t*-test was used to calculate the *p*-value for each metabolite when variances between groups were unequal. Given the large number of metabolites tested, the Benjamini-Hochberg (BH) procedure was applied to control the false discovery rate (FDR). Metabolites were considered significantly differential between groups when they simultaneously satisfied the following criteria: VIP (OPLS-DA) > 1 and adjusted *p* < 0.05. For KEGG pathway enrichment analysis, *p*-values were corrected using the BH method, and pathways with a corrected *p*-value < 0.05 were considered significantly enriched. All statistical analyses were performed using the Majorbio Cloud Platform and R software.

### 4.17. Metabolome and Intestinal Microbiota Correlation Analysis

To explore the potential associations between key metabolites and gut microbial taxa, Spearman’s rank correlation analysis was performed using the Majorbio Cloud Platform and R software, with the correlation matrix between significantly differential metabolites and dominant bacterial groups being calculated. Given the large number of metabolite–phylum level gut microbiota, *p* values were adjusted for multiple testing using the BH method. Correlations with an adjusted *p* value < 0.05 were considered statistically significant. The results were presented as heatmaps, with significant associations indicated.

### 4.18. Statistical Analysis

All data were expressed as mean ± standard deviation (SD). Differences between two groups were analyzed using Student’s *t*-test, while comparisons among multiple groups were performed using one-way analysis of variance (ANOVA) followed by the Tukey–Kramer test. All statistical analyses were conducted using GraphPad Prism 8.5 (GraphPad Software, La Jolla, CA, USA). *p* < 0.05 was considered statistically significant.

## 5. Conclusions

In this study, administration of EgHCF alleviated colitis symptoms in DSS-induced UC mice, suppressed the inflammatory response, restored impaired intestinal barrier function, protected the colon from oxidative damage, and activated the Keap1/Nrf2 signaling pathway. Furthermore, EgHCF increased gut microbial diversity, ameliorated gut dysbiosis, and regulated tryptophan metabolism. Taken together, these findings suggest that EgHCF plays an important role in protecting against ulcerative colitis. However, given that this study was conducted solely in a mouse model, the protective efficacy of EgHCF in patients with ulcerative colitis has yet to be established. In addition, because the present study employed an acute DSS-induced UC model, the current results are insufficient to evaluate the long-term safety and protective effects of EgHCF. Accordingly, future studies will establish a DSS-induced chronic colitis model, or other models of slowly progressive autoimmune diseases, to more comprehensively assess the protective effects of EgHCF against colitis.

## Figures and Tables

**Table 1:** Disease activity index (DAI) scoring criteria.

Score	Weight Loss (%)	Stool Consistency	Occult Blood or Bloody Stool
No change	0	Normal	Negative
1	1–5%	Mid soft	Negative
2	6–10%	Soft and wet	Occult Blood
3	10–20%	Half loose stool	Bloodstains can be seen in the feces
4	>20%	Loose stool	Rectal bleeding

**Table 2:** Primer sequences used for qRT-PCR.

Gene	Primer	Reference Sequence
GCLM	Sense: 5′-GCCTTTGCTAAACAATTTGACA-3′	CT010256.1
	Anti-sense: 5′-AGTACCTCAGCAGCCACAGC-3′	
HO-1	Sense: 5′-ACCTTCCCGAACATCGACAGC-3′	NM_010442.2
	Anti-sense: 5′-AATGTTGAGCAGGAAGGCGG-3′	
NQO1	Sense: 5′-CCTCAACTGGTTTACAGCATTG-3′	CT010284.1
	Anti-sense: 5′-GGTCAAACAGGCTGCTTGGA-3′	
GCLC	Sense:5′-GGCCACTATCTGCCCAATTG-3′	XM_006510812.2
	Anti-sense: 5′-CCTCCCGTGTTCTATCATCTAC-3′	
β-actin	Sense: 5′-GGCTGTATTCCCCTCCATCG-3′	BC138614.1

## Data Availability

The original contributions presented in this study are included in the article. Further inquiries can be directed to the corresponding author.

## Supplementary Materials

The following supporting information can be downloaded at: https://www.mdpi.com/article/10.3390/ijms27209286/s1.

## Abbreviations

The following abbreviations are used in this manuscript:

Eg	*Echinococcus granulosus*
HCF	Hydatid cyst fluid
UC	Ulcerative colitis
DSS	Dextran Sulfate Sodium Salt
IBD	Inflammatory bowel disease
5-ASA	5-aminosalicylic acid
SCFAs	Short-Chain Fatty Acid
CE	Cystic echinococcosis
LL	Laminated Layer
GL	Germinal Layer
PSC	Protoscoleces
TJs	Tight junctions
MPO	Myeloperoxidase
IHC	Immunohistochemistry
IF	Immunofluorescence
DCFH-DA	2′,7′-Dichlorodihydrofluorescein diacetate
SDS-PAGE	Sodium Dodecyl Sulfate Polyacrylamide Gel Electrophoresis
