# Supplementary material for: Hydatid Cyst Fluid of Echinococcus granulosus Alleviates Experimental Ulcerative Colitis by Suppressing Inflammation and Oxidative Stress and Regulating the Gut Microbiota
Source: Int J Mol Sci. 2026 Oct 19;27(20):9286. doi: 10.3390/ijms27209286 (PMC13705532; doi:10.3390/ijms27209286)
Supplement: Supplementary file 1 [file ijms-27-09286-s001.zip › ijms-4474796-supplementary.pdf]

Supplementary Figure

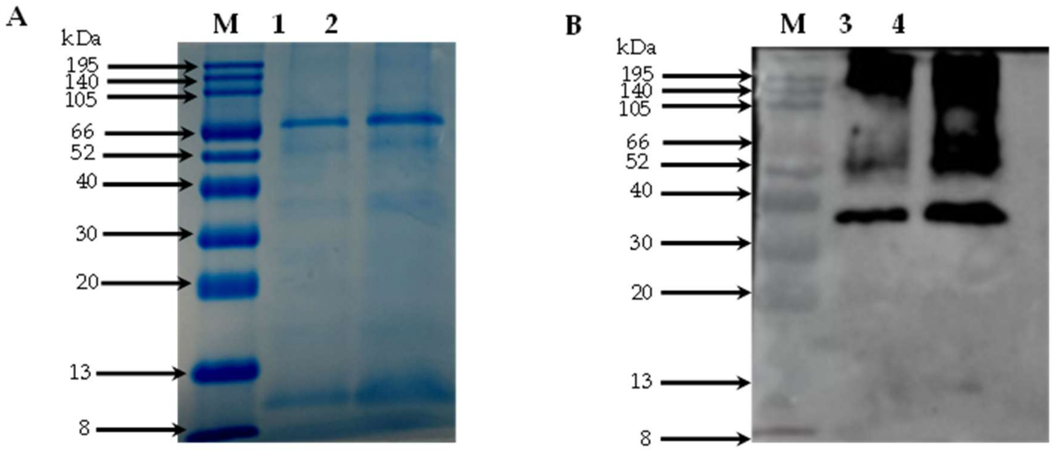

Figure S1. Protein profiling and immunogenicity of *E. granulosus* hydatid cyst fluid (EgHCF). (A) EgHCF (20 μg per lane) was separated by 10% SDS-PAGE. M: Protein Marker (8-195kDa). Lanes 1-2: EgHCF. (B) Western blot analysis of EgHCF. M: Protein Marker (8-195kDa) . Lanes 3 – 4: EgHCF probed with anti-EgHCF mouse IgG.

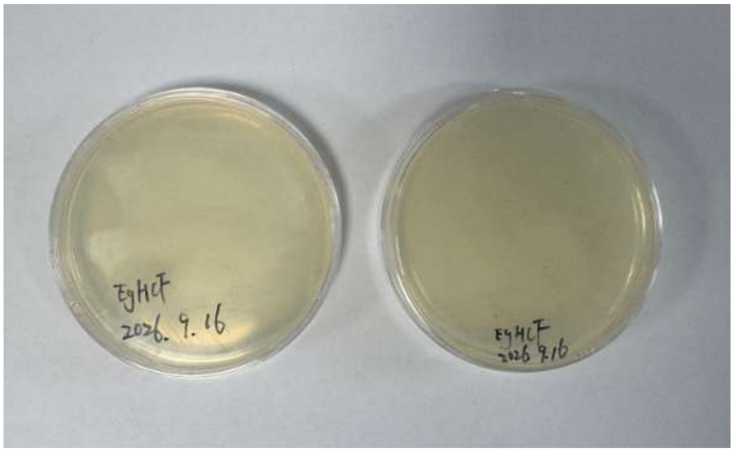

Figure S2. Bacterial contamination test: an antibiotic-free LB solid medium was used to assess for contamination of EgHCF.
